# An *Lmx1b-miR135a2* Regulatory Circuit Modulates *Wnt1*/Wnt Signaling and Determines the Size of the Midbrain Dopaminergic Progenitor Pool

**DOI:** 10.1371/journal.pgen.1003973

**Published:** 2013-12-12

**Authors:** Angela Anderegg, Hsin-Pin Lin, Jun-An Chen, Giuliana Caronia-Brown, Natalya Cherepanova, Beth Yun, Milan Joksimovic, Jason Rock, Brian D. Harfe, Randy Johnson, Rajeshwar Awatramani

**Affiliations:** 1 Northwestern University Feinberg School of Medicine, Department of Neurology and Center for Genetic Medicine, Chicago, Illinois, United States of America; 2 Institute of Molecular Biology, Academia Sinica, Taipei, Taiwan; 3 Department of Molecular Genetics and Microbiology, University of Florida, Gainesville, Florida, United States of America; 4 Department of Biochemistry and Molecular Biology, The University of Texas M.D. Anderson Cancer Center, Houston, Texas, United States of America; Sloan Kettering Institute, United States of America

## Abstract

MicroRNAs regulate gene expression in diverse physiological scenarios. Their role in the control of morphogen related signaling pathways has been less studied, particularly in the context of embryonic Central Nervous System (CNS) development. Here, we uncover a role for microRNAs in limiting the spatiotemporal range of morphogen expression and function. Wnt1 is a key morphogen in the embryonic midbrain, and directs proliferation, survival, patterning and neurogenesis. We reveal an autoregulatory negative feedback loop between the transcription factor Lmx1b and a newly characterized microRNA, *miR135a2*, which modulates the extent of *Wnt1*/Wnt signaling and the size of the dopamine progenitor domain. Conditional gain of function studies reveal that *Lmx1b* promotes *Wnt1*/Wnt signaling, and thereby increases midbrain size and dopamine progenitor allocation. Conditional removal of *Lmx1b* has the opposite effect, in that expansion of the dopamine progenitor domain is severely compromised. Next, we provide evidence that microRNAs are involved in restricting dopamine progenitor allocation. Conditional loss of *Dicer1* in embryonic stem cells (ESCs) results in expanded Lmx1a/b+ progenitors. In contrast, forced elevation of *miR135a2* during an early window *in vivo* phenocopies the *Lmx1b* conditional knockout. When *En1::Cre*, but not *Shh::Cre* or *Nes::Cre*, is used for recombination, the expansion of Lmx1a/b+ progenitors is selectively reduced. Bioinformatics and luciferase assay data suggests that *miR135a2* targets *Lmx1b* and many genes in the Wnt signaling pathway, including *Ccnd1, Gsk3b, and Tcf7l2*. Consistent with this, we demonstrate that this mutant displays reductions in the size of the *Lmx1b/Wnt1* domain and range of canonical Wnt signaling. We posit that microRNA modulation of the Lmx1b/Wnt axis in the early midbrain/isthmus could determine midbrain size and allocation of dopamine progenitors. Since canonical Wnt activity has recently been recognized as a key ingredient for programming ESCs towards a dopaminergic fate *in vitro*, these studies could impact the rational design of such protocols.

## Author Summary

To achieve exquisitely complex behavior, the mammalian CNS is comprised of numerous neuron types, each with different functions. These distinct neuron types are produced from neural progenitors during embryonic development. How the embryonic neural progenitors are programmed to produce distinct neuron types, in the correct position and number, is a central question in developmental neuroscience. We focused on studying the embryonic production of a key neuron type, the midbrain dopamine neuron (mDA), which is particularly vulnerable in Parkinson's disease (PD). Previous works from our lab and others have shown that Wnt signaling is critical for dopamine neuron production. Here we provide a mechanism for how Wnt signaling is initiated, and then downregulated. Key to initiating this process is a transcription factor, Lmx1b, whereas important to the downregulation process is a newly characterized microRNA, *miR135a2*. The quantitative balance of these factors determines how many dopamine neurons are produced during embryonic development. These studies will have direct implications for efficiently programming dopamine neurons from stem cells, a key goal of regenerative approaches for PD.

## Introduction

MicroRNAs regulate gene expression in various aspects of central nervous system (CNS) and peripheral nervous system (PNS) development and function, including neurogenesis, glial differentiation, fate specification, synaptogenesis, spine formation and plasticity [1]–[7]. Less studied is their role in modulating the most critical developmental signaling molecules in the embryonic CNS – morphogens. Recent studies have suggested that morphogen function is not simply based on a concentration gradient, but rather an integral of concentration as well as the time of exposure [8]–[10]. Thus, mechanisms must exist to control the dose and time of morphogen expression and function. MicroRNAs have been shown to target key elements of morphogen pathways in the early embryo [11]. We considered it plausible that microRNAs may be involved in modulating morphogen function in the developing CNS.

Wnts are key morphogens in the developing and adult CNS that are involved in proliferation, survival, patterning, and neurogenesis [12]–[14]. Wnt1 is the prototypical canonical Wnt and its function has been documented particularly in the midbrain region [14]. *Wnt1* is dynamically expressed in the midbrain, being expressed in a broad swath at 8.5 days post coitum (dpc) and ultimately restricting to the Roof Plate (RP), Isthmic Organizer (IsO) and Floor Plate (FP) regions. Loss of *Wnt1* leads to a drastic decrease in midbrain size, as well as reduction and misspecification of midbrain dopamine neurons (mDAs), and this is exacerbated by loss of *Wnt5a* [15], [16]. Studies that have interrupted Wnt/beta-catenin signaling have revealed that this pathway is critical for specification and neurogenesis of mDAs [17]–[19]. Wnt signaling is required for the expression of key mDA determinants Lmx1a, Otx2, and Ngn2 and for the downregulation of Shh [17]. Counterintuitively, excessive Wnt signaling is also detrimental for mDA production [20], adding to the general notion that morphogen dosage must be tightly regulated [21].

In the ventral midbrain, the Foxa2+, Shh+ FP is roughly allocated into two main progenitor domains: a medial Lmx1a/b+/Msx+ progenitor domain that gives rise to many mDAs (this domain may be further subdivided [22], [23]), and a lateral Nkx6.1+/Sim1+ domain that gives rise to many Brn3a+ neurons, populating among others, the red nucleus [22], [24]–[28]. During early development, however, Nkx6.1 is expressed at the midline and Lmx1b is expressed more broadly than Lmx1a/Msx. Between 9.0–9.5 dpc, Lmx1a/Msx expression is initiated, and expands laterally [27]. At the midline, Lmx/Msx ultimately subsumes Nkx6.1, resulting in a medial Lmx/Msx and lateral Nkx6.1/Sim1 domain demarcated by a sharp boundary. Lineage-based progenitor labeling studies have suggested that the expansion of the Lmx1a domain, in part occurs by inductive mechanisms [22], [28]. This induction is likely, at least in part, mediated by Wnt1/beta-catenin signaling, which is important in the ventral midbrain [17], [29]–[31], and both necessary and sufficient for Lmx1a expression in the FP [17], [18], [32]. Indeed, this capacity to elicit drastic gene expression changes makes the ventral midbrain a particularly good model to interrogate *Wnt1*/Wnt signaling and its modulators.

In this study we have identified an autoregulatory loop involving *Lmx1b* and *miR135a2* that is critical for determining mDA allocation. We show that *Lmx1b* promotes mDA progenitor fate, whereas *miR135a2* delimits the mDA domain. Forced maintenance of *Lmx1b* results in expanded mDA progenitors whereas loss of *Lmx1b* results in diminished mDA progenitors. MicroRNA studies show the opposite effects. Conditional removal of *Dicer1* from ESCs results in expanded mDA progenitors at the expense of Nkx6.1 progenitors. In contrast, increased *miR135a2* levels, only during an early window, result in a reduction in the proportion of mDA progenitors. In addition to progenitor allocation defects, we observed changes in midbrain size in these mutants. Both progenitor allocation and midbrain size phenotypes may be caused, at least in part, by alterations in *Wnt1*/Wnt signaling. While *Lmx1b* promotes *Wnt1*/Wnt signaling, *miR135a2* appears to negatively regulate *Lmx1b*/*Wnt1*/Wnt signaling in the context of the embryonic midbrain.

## Results

### *miR135a2* is enriched in the ventral midbrain and embedded within a novel intron of *Rmst*

**Figure 1:**
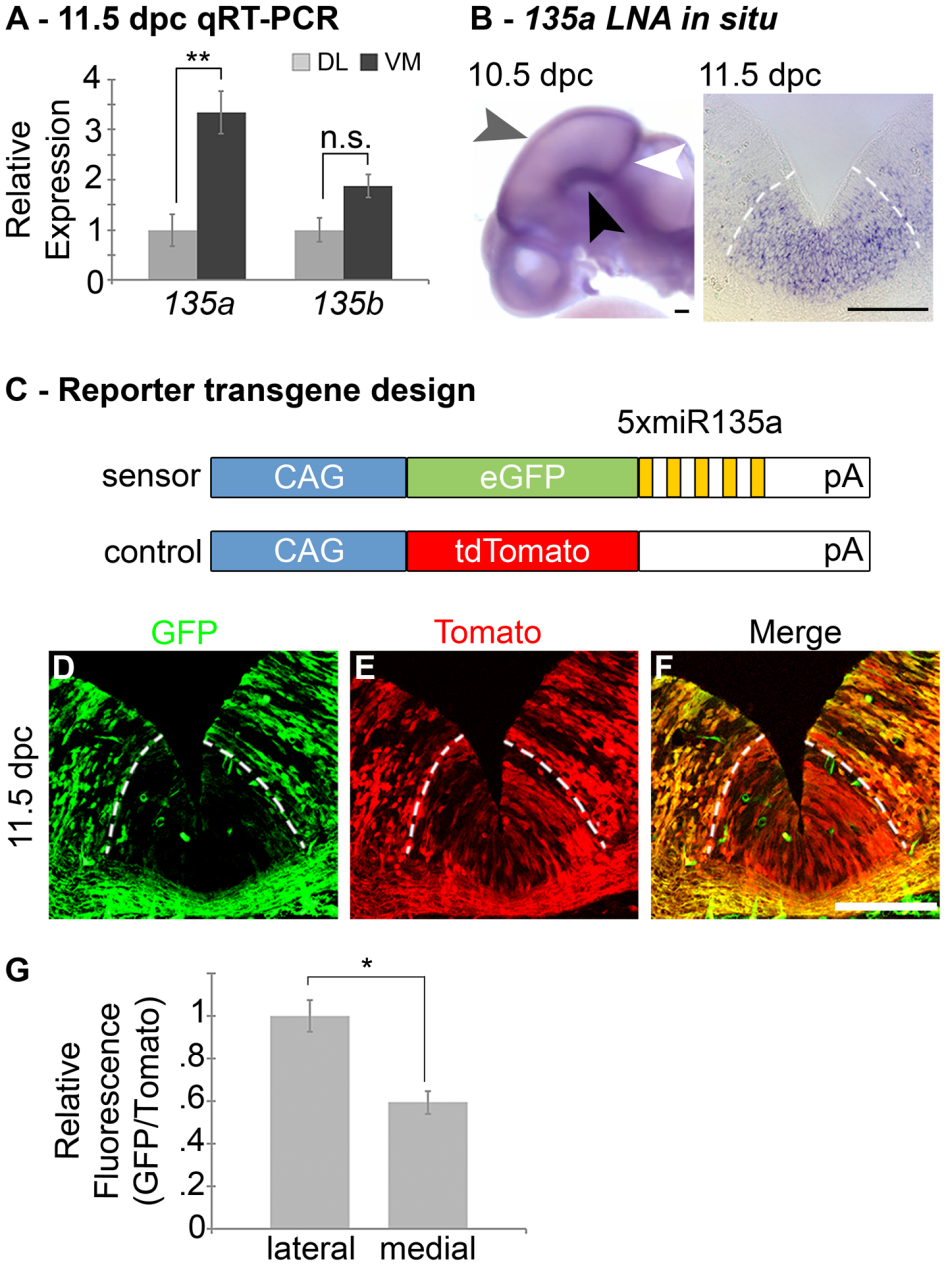
Expression of *miR135a* within the *Wnt1*-rich ventral midbrain. (A) Quantitative Real-time RT-PCR on 11.5 dpc dorsolateral (DL) and ventromedial (VM) midbrain regions revealed that *miR135a* is enriched in the ventral midbrain (n = 3; DL mean = 1±.08, VM mean = 3.26±.05; p-value = 0.01). The increase in closely related *miR135b* is not statistically significant (n = 3; DL mean = 1±.06, VM mean = 1.38±.05; p-value = 0.16). (B) Locked nucleic acid (LNA) *in situ* hybridization at 10.5 dpc showed elevated *miR135a* in the midbrain FP (black arrowhead), RP (grey arrowhead), and IsO (white arrowhead). Coronal sections at 11.5 dpc show enrichment in ventral midline progenitors, the same region where *Wnt1* is expressed. (C) Schematic of control and “sensor” transgene to detect *miR135a* activity. (D–F) 11.5 dpc coronal sections from transient double transgenic embryos revealed severe downregulation of the eGFP reporter, which contained several *miR135a* binding sites, and little to no downregulation of the tdTomato reporter, which contained no binding sites. 12.5 dpc embryos showed a similar pattern (n = 4; data not shown). (G) Ratios of confocal line scan measurements for GFP and Tomato intensity in lateral (outside of the *135a LNA* expression domain indicated by dotted white lines) and medial (within the *135a LNA* expression domain) regions reveal a 42% reduction in GFP expression medially at 11.5 dpc (n = 3; lateral mean = 1±.09, medial mean = .58±.07; p-value = .02). Scale bars represent 100 µM.

In order to identify microRNAs (miRs) in the *Wnt1*–rich mDA progenitor domain, ventral midline and dorsal lateral tissues were microdissected from 11.5 dpc mouse embryos and used to perform a microRNA array, followed by qRT-PCR validation with select individual TaqMan assays (Applied Biosystems). We reasoned that functionally relevant microRNAs would be 1) robustly expressed 2) differentially expressed and 3) bioinformatically predicted to target genes in the Lmx/Wnt axis. From five candidates that fit these criteria, we focused on *mmu-miR-135a (miR135a)*, which was robustly expressed and showed a greater than 3-fold increase in the ventral midline compared to dorsal lateral tissue. The closely related *mmu-miR-135b (miR135b)* was also increased in the ventral midbrain, but this increase was not statistically significant (Figure 1A). Ventral midbrain enrichment of *miR135a* was further confirmed by Locked Nucleic Acid (LNA, Exiqon) *in situ* hybridization, which is designed to specifically detect mature microRNAs (Figure 1B). In addition, *miR135a* was predicted to target *Lmx1b* and several genes of the Wnt pathway through evolutionarily conserved binding sites in the *3′UTR* [33](Table 1), thereby warranting further study.

**Table 1:** Predicted *miR135a* targets in the Wnt signaling pathway.

Gene	TargetScan	miRanda (microrna.org)
Apc		Apc
Axin1,2		Axin1
B-arrestin (Arrb1,2)		Arrb1
Ccnd1		Ccnd1
CK1 (casein kinase 1 - Csnk1)	Csnk1a1, g1	Csnk1, g1
Ctgf/Cyr61		Ctgf
Dishevelled homologs (1, 2, 3)	Dvl1	Dvl1
Dkk homologs (1, 2, 3, 4)		Dkk2
Frizzled homologs	Fzd1, Fzd3	Fzd1, Fzd10, Fzd4,
Gsk3b	Gsk3b	Gsk3b
Klhl12		Klhl12
Kremen1, 2	Kremen1	
Lrp5/6	Lrp6	
Nkd1	Nkd1	
Nlk		Nlk
Norrin (Ndp)	ndp	Ndp
PP1 (Ppp1cc)	Ppp1cc	Ppp1cc
PR61 (Ppp2r5c)	Ppp2r5c	Ppp2r5c
PRR (Pvrl1)	Pvrl1	Pvrl1
Ranbp3	Ranbp3l	
Ror2	Rorb	
R-spondin homologs (Rspo1, 2, 3, 4)	Rspo2	Rspo2, 3, 4
Sfrp1, 2, 3, 4, 5		Sfrp2
Shisa homologs	Shisa2, 6, 7, 9	Shisa2, 5, 6, 7, 9
TCF/LEF	Tcf7, Tcf7l2,Tcf23, Hnf1a, Hnf4a, Nfe2l1, Tead1, Zeb1, Zfp354a	Tcf4, Tcf7, Tcf7l2,Tcf15, Tcf23, Hnf1a, Hnf4a, Nfe2l1, Tead1, Zeb1, Zfp354a
VPS4	Vps4a	Vps4a
Wnts	Wnt3, Wnt5a, Wnt9a, Wnt9b, Wnt10b	Wnt1, Wnt3, Wnt5a, Wnt8a, Wnt9a, Wnt9b, Wnt10b
**Total Targets:**	**35**	**47**

A large number of genes involved in canonical Wnt signaling (including ligands, receptors and downstream transcriptional regulators) are bioinformatically predicted targets of *miR135a* by TargetScan and miRanda. A handful of genes (*Drapc1, Ctnnb1, Cav1, Cul3, Cxxc4, Dact1, Frat2, Lmna, Macf1, Pas5a, Porcn, Ppp2ca, Sost, Sostdc1, Wif1 and Wls*) were not found to be targets by these algorithms. This result suggests that *miR135a2* likely acts through multiple levels of the Wnt cascade to modulate Wnt signaling.

To extend the expression data, we designed a reporter transgene (“sensor”) to verify the functional activity of *miR135a* in the ventral midbrain [34]. This transgene, comprised of *eGFP* with several sequences complementary to *miR135a* in the *3′UTR*, is designed to broadly express eGFP. In regions of high *miR135a* activity, however, the eGFP levels should be suppressed. Since we did not design bulges in the microRNA binding sites, this transgene will not serve as a microRNA “sponge”, but only as a “sensor”. A control transgene, comprised of *tdTomato* with no complementary *miR135a* sites in the *3′UTR*, was designed to broadly express tdTomato regardless of microRNA activity (Figure 1C). The transgenes were co-injected and transient transgenic embryos were harvested at 11.5 or 12.5 dpc. In ventral midline progenitors, eGFP was markedly reduced in the region predicted to have high *miR135a* activity, equivalent to that detected by the *135a LNA* probe. In contrast, tdTomato showed little to no reduction at the ventral midline compared to neighboring regions (Figure 1D–G).

**Figure 2:**
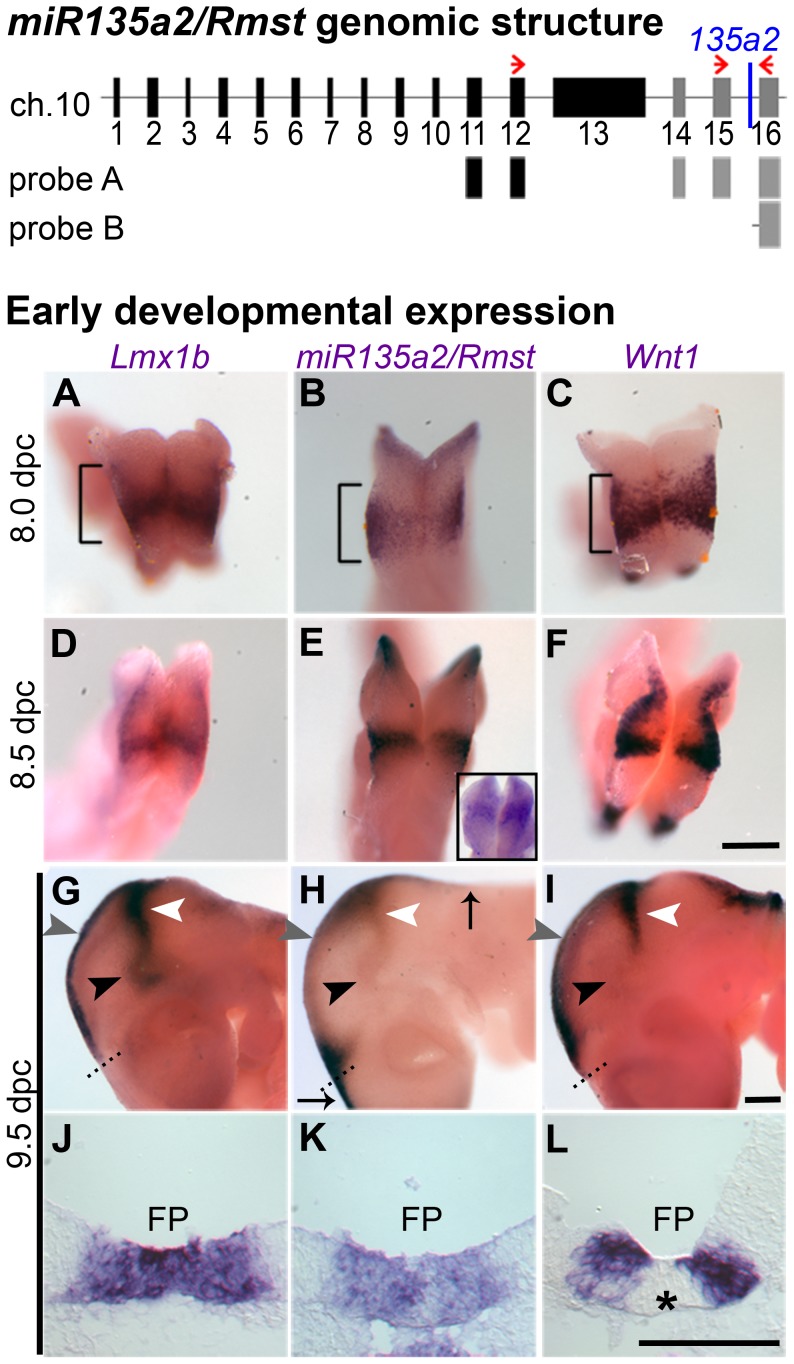
Identification of *miR135a2* within an intron of a transcript that is expressed in a manner similar to *Lmx1b* and *Wnt1* in the midbrain. (Top) Proposed organization of the *miR135a2/Rmst* transcript. Using two different primer sets (red arrows) for RT-PCR on 11.5 dpc ventral midbrain, we revealed that the known *Rmst* transcript (black boxes) can be extended by three additional exons (grey boxes) and *miR135a2* is embedded in the final detected intron. Two *in situ* probes designed to this region, *probe A* and *probe B*, show expression similar to the *135a LNA probe* (see Figure S1B). (Bottom, A–L) Whole mount *in situs* showed widespread *Lmx1b*, *miR135a2/Rmst*, and *Wnt1* in the prospective midbrain (brackets) at early time points. The *135a LNA probe* (shown as an inset in panel E) had similar widespread expression. The expression of these genes was restricted to midbrain FP (black arrowhead), RP (grey arrowhead) and IsO (white arrowhead) by 9.5 dpc. Dorsally, *miR135a2/Rmst* hybridization extended into the hindbrain and telencephalon (black arrows). Coronal sections further demonstrate the tightly correlated FP expression of all three probes, although *Wnt1* is excluded from the midline (asterisk), likely due to as yet uncharacterized repressors. Scale bars represent 100 µM.

In mice, there are two *miR135a* family members, *mmu-miR135a-1* (*miR135a1*) and *mmu-miR135a-2* (*miR135a2*). Below we provide evidence suggesting that *miR135a2*, is expressed in the midbrain. Although *miR135a2* was predicted to be intergenic on the miRBase Sequence Database and UCSC genome browser, a separate screen [35] coupled with further bioinformatic analysis revealed that *miR135a2* was likely located between two exons of a previously uncharacterized gene. Based on its proximity to the 3′ end of nearby non-coding RNA, *Rhabdomyosarcoma 2 associated transcript* (*Rmst*)(Ensembl browser), which is known to be expressed in the midbrain [36], we hypothesized that *miR135a2* was embedded in this gene. Thus, we performed RT-PCR on 11.5 dpc ventral midbrain RNA using a forward primer in *Rmst* and a reverse primer in the downstream flanking exon of *miR135a2*. This experiment yielded two predominant bands of approximately 700 bp and 900 bp (Figure S1A), indicating possible splice variants that will be further characterized in a future study. The most prominent fragment was sequenced and a BLAST search revealed that a) a variant of the *Rmst* transcript exists, which excludes exon 13, and has at least three additional exons and b) *miR135a2* is located in the final detected intron of this transcript (Figure 2 – top panel). Moreover, *in situ* hybridizations with two separate probes (*probe A* and *probe B*) designed against this region showed similar expression in the midbrain and hindbrain FP, RP from the hindbrain to the telencephalon, and the IsO (Figure 2H; Figure S1B), equivalent to that detected by the *135a LNA probe* (Figure 1B). Together, these results suggest that *miR135a2* is coexpressed with *Rmst* in the midbrain. Although a separate internal promoter for the microRNA remains an alternative possibility, it is likely processed from an intron, a finding common for more than 50% of microRNAs [37].

### *miR135a2/Rmst* expression is dynamic and resembles that of *Lmx1b* and *Wnt1*

**Figure 3:**
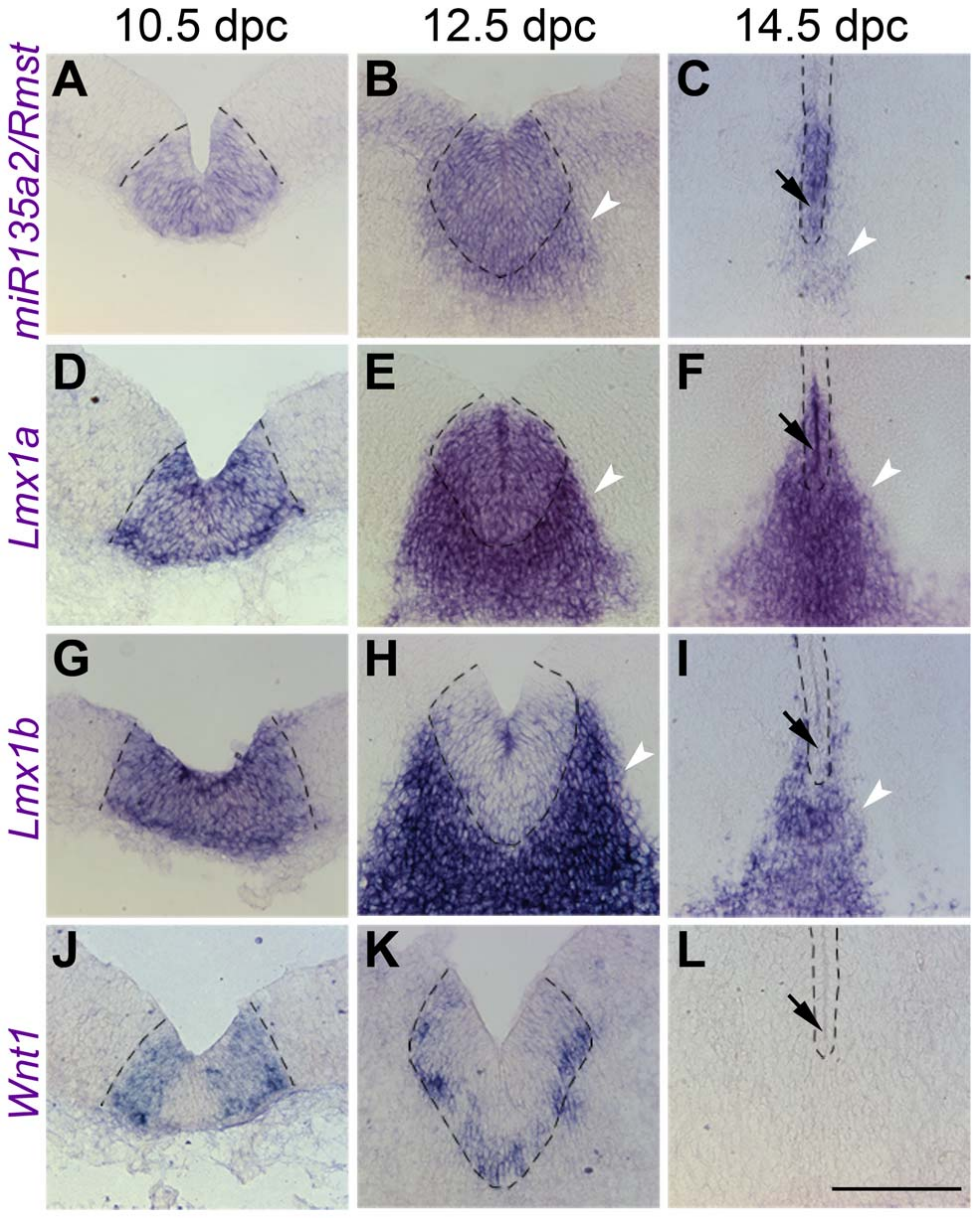
Temporal exclusion of *miR135a2/Rmst* and *Lmx1b/Wnt1* in mDA progenitors. (A–C) Coronal section *in situ* hybridization experiments show that *miR135a2/Rmst* is mainly expressed within the mDA progenitor domain (dotted black lines) between 10.5 dpc and 14.5 dpc, though there is some expression in newly postmitotic cells (white arrowheads). (D–F) *Lmx1a* is similarly maintained in mDA progenitors, and may contribute to maintenance of *miR135a2/Rmst* expression within this domain at later stages. (G–L) On the other hand, *Lmx1b* and *Wnt1* are robustly expressed at 10.5 dpc, downregulated by 12.5 dpc and drastically reduced from the ventricular zone by 14.5 dpc. The mutual exclusion seen within the ventricular zone at later stages (black arrows in C, F, I, and L) suggests a role for *miR135a2/Rmst* in temporally restricting the expression of *Lmx1b/Wnt1*, but not that of *Lmx1a*. Scale bar represents 100 µM.

The presence of *miR135a2/Rmst* in the FP, RP, and IsO resembles previously described expression patterns of two important genes, *Lmx1b* and *Wnt1* [38], [39]. Thus, we compared the spatio-temporal relationship of these genes throughout early midbrain development. We observed similar and widespread expression with all three probes, as well as the *miR135a LNA* probe at 8.0 dpc, likely in the prospective midbrain, based on pan-midbrain reporter expression in *Wnt1::Cre* fate maps (Figure S1C). By 9.5 dpc all three genes were no longer detected throughout the midbrain, but rather were restricted to the FP, RP, and IsO (Figure 2A–L). The expression of these three genes correlates until ∼11.5 dpc, but at this age *miR135a2/Rmst* is also observed, at lower levels, in cells appearing to exit the ventricular zone throughout the midbrain (Figure S1B). This expression likely contributes to the low levels of *miR135a* observed in the dorsolateral samples in Figure 1A and is likely independent of *Lmx1b*. Between 12.5 dpc and 14.5 dpc, the microRNA transcriptional unit is maintained in dopamine progenitors, whereas *Lmx1b* and *Wnt1* are severely downregulated, consistent with the phenomenon of “temporal exclusion” that has been described for many microRNAs and their cognate targets (Figure 3) [40]. On the other hand, *Lmx1a*, which is closely related to, and has partially overlapping function with *Lmx1b*, is not an *in silico* predicted target of *miR135a2* and remains easily detectable in dopamine progenitors (Figure 3D–F).

These dynamic and tightly correlated expression patterns suggested *miR135a2/Rmst* as a potential component of the *Lmx1b*/*Wnt1* regulatory network. At this point, we designed experiments to elucidate the details of this network by testing a) the hierarchical relationship between *Lmx1b*, *miR135a2/Rmst*, and *Wnt1* b) the role of *Lmx1b* in midbrain development, and c) whether microRNAs, and specifically *miR135a2* levels, were important for early midbrain development.

### Lmx1b is necessary and sufficient for normal *Wnt1*/Wnt signaling and *miR135a2/Rmst* expression in the midbrain

**Figure 4:**
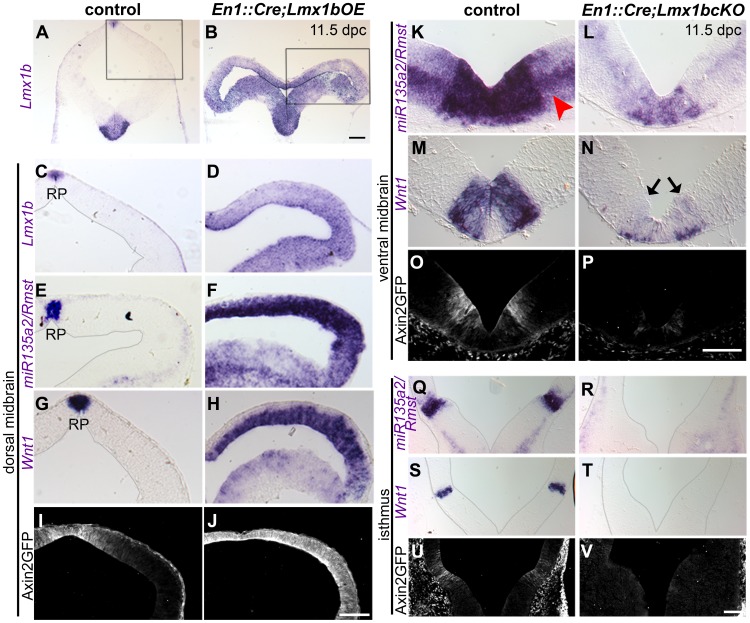
*Lmx1b* is necessary and sufficient for normal expression of *miR135a2/Rmst, Wnt1*, and Wnt signaling in the midbrain. (A–H) In *En1::Cre;Lmx1bOE* embryos, *in situ* hybridization at 11.5 dpc shows that *Lmx1b, mir135a2/Rmst*, and *Wnt1* are ectopically expressed throughout the midbrain progenitor zone, albeit at varying levels. (I–J) The expression of the *Axin2::d2eGFP* transgene was observed throughout the midbrain in mutant embryos, but not in controls (control sections in C, E, and G were outlined to accentuate tissue; black boxes in A and B indicate region of high magnification in C–J). (K–P) 11.5 dpc *En1::Cre;Lmx1bcKO* embryos show reduced expression of *miR135a2/Rmst* (red arrowhead points to weaker expression of *miR135a2/Rmst* outside the dopamine progenitor domain), *Wnt1* (black arrows indicate remnant *Wnt1* expression), and d2eGFP in the dopamine progenitor domain. (Q–V) These mutants show nearly complete loss of *miR135a2/Rmst*, *Wnt1* and d2eGFP in the isthmus (sections in Q-T were outlined to accentuate tissue). Scale bars represent 100 µM.

Given the overlapping expression patterns of *Lmx1b*, *miR135a2/Rmst* and *Wnt1* in the FP, RP and IsO, we first tested the hierarchical relationship between these genes. To do this, we utilized a mouse strain designed to conditionally express *Lmx1b* coding sequence, but lacking greater than 95% of the *3′UTR*, under control of robust *CAG* regulatory elements. Using this strain, we generated embryos in which *Lmx1b* was conditionally activated throughout the midbrain and rhombomere 1 from ∼8.0 dpc onward, with *Engrailed 1 (En1) Cre* recombination [41], [42]. Forced maintenance of Lmx1b in *En1* ^Cre/+^; *Rosa26^Lmx1b/+^* (*En1::Cre;Lmx1bOE*) led to ectopic *mir135a2/Rmst* and *Wnt1* expression in the midbrain, although both of these *mRNAs* were expressed more robustly in dorsal compared to ventral regions (Figure 4A–H). To determine whether changes in *Wnt1* expression correlated with changes in Wnt signaling, we employed an *Axin2::d2eGFP* reporter allele that provides a transcriptional readout of this pathway [43], [44]. In *En1::Cre;Lmx1bOE,Axin2::d2eGFP* embryos d2eGFP fluorescence is detectable throughout the midbrain, whereas in controls it is predominantly confined to the ventral midline and the region surrounding the roof plate (Figure 4I–J and data not shown).

Conversely, in embryos wherein *Lmx1b* was conditionally deleted (*En1::Cre;Lmx1bcKO*) [45], *miR135a2/Rmst* and *Wnt1* were significantly reduced in mDA progenitors (Figure 4K–N), undetectable in the isthmus (Figure 4Q–T), and mildly reduced in the dorsal midbrain (Figure S2A–D). *Axin2::d2eGFP* transgene expression was drastically reduced in the ventral midbrain (Figure 4O–P) and barely detectable in the isthmus (Figure 4U–V), but appeared only slightly diminished in the dorsal midbrain (Figure S2E–F). These data suggest that Lmx1b is upstream of both genes and promotes Wnt signaling, but that in regions where Lmx1a is expressed, it can at least partially compensate for Lmx1b. This finding is consistent with *in vitro* studies wherein both Lmx1a and Lmx1b were shown to drive *Wnt1* expression [46].

Finally, to determine whether the observed changes in *miR135a2/Rmst* corresponded to the mature microRNA, we used qRT-PCR to quantify mature *miR135a* levels. In *En1::Cre,Lmx1bOE* midbrain, mature microRNA was increased 2-fold, whereas in *En1::Cre,Lmx1bcKO* midbrain it was modestly reduced (Figure S2G). These results suggest that *miR135a2* is coexpressed with the *Rmst* transcript and responsive to *Lmx1b* manipulations.

### Lmx1b determines the size of the midbrain, the FP, and the mDA progenitor domain

**Figure 5:**
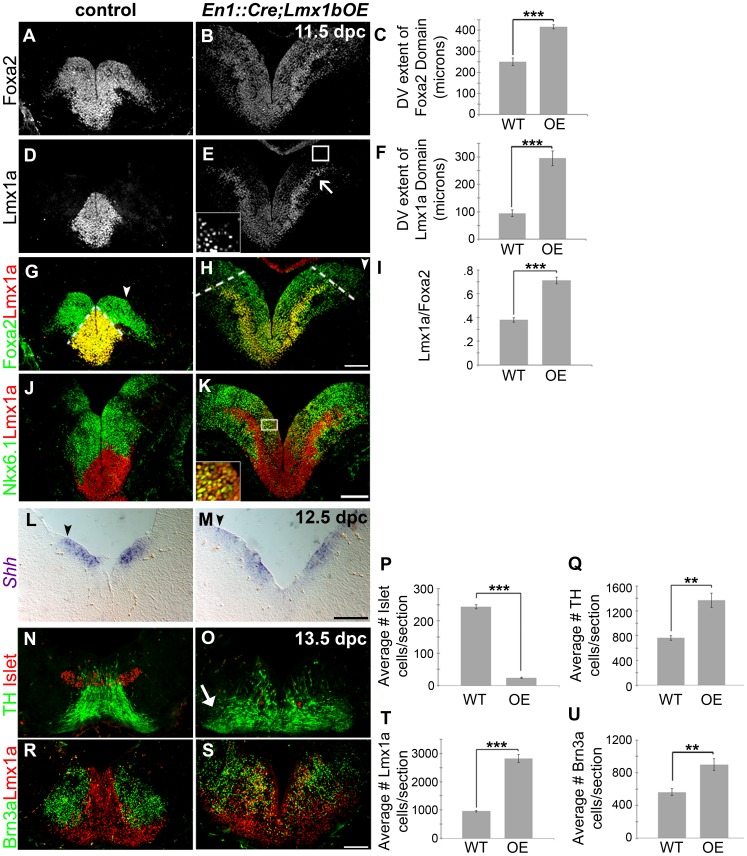
Maintenance of *Lmx1b* alters the size of the FP, the mDA progenitor pool, and ventral midbrain patterning. (A–K) Immunolabeling of 11.5 dpc *En1::Cre;Lmx1bOE* embryos revealed the dorsal expansion of Foxa2 and Lmx1a progenitor domains, although per cell levels of both markers were reduced. The closed white arrowheads indicate the dorsal border of Foxa2 expression, graphically represented in C (n = 3; control mean = 251.27±18.20 µM, mutant mean = 416.19±9.20 µM; p-value = 0.855E-05). The dotted white lines indicate the dorsal border of Lmx1a expression, graphically represented in F (n = 3; control mean = 94.67±11.73 µM, mutant mean = 295.30±26.58 µM; p-value = 0.0002), and as a normalized ratio in I (p-value = 0.0006) (white box in E surrounds the border region of Lmx1a progenitors, which is shown at a higher magnification and exposure in the inset). Ectopic Lmx1a+ neurons appear to emanate from the lateral Foxa2 regions (open arrow). In these lateral regions, a mixed Lmx1a+/Nkx6.1+ progenitor pool was observed (white box in K surrounds region shown in inset). (L–M) At 12.5 dpc *Shh* expression was also expanded dorsally (closed black arrowheads indicate the dorsal border of expression). (N–U) In 13.5 dpc *En1::Cre;Lmx1bOE* embryos we found a 90% reduction in Islet+ oculomotor neurons (n = 3; control mean = 243.57±6.33, mutant mean = 23.58±2.01; p-value = 1.66E-05), an 80% increase in Brn3a+ red nucleus neurons (n = 3; control mean = 562.33±34.59, mutant mean = 901.17±59.47; p-value = .008), a 194% increase in Lmx1a+ cells (n = 3; control mean = 958.22±26.04, mutant mean = 2817.11±114.16; p-value = 9.20E-05) and an 80% increase in TH+ mDAs (n = 3; control mean = 763.33±30.62, mutant mean = 1370.89±93.01; p-value = .003). Ectopic TH+ neurons were particularly observed in more lateral regions (closed arrow). Some post-mitotic cells were found to be mixed phenotype in that they were both Lmx1a+ and Brn3a+. Scale bars represent 100 µM.

To determine the role of Lmx1b in midbrain development, we first investigated the functional consequences of maintaining *Lmx1b* in the early embryonic midbrain. Coronal sections through *En1::Cre;Lmx1bOE* embryos revealed an overall increase in third ventricle size and morphogenetic abnormalities (Figure S3A–B, G–H). In the ventral midbrain of 9.5–11.5 dpc *En1::Cre;Lmx1bOE* embryos we observed the dorsal-ventral (DV) extent of the FP marker Foxa2 to be significantly expanded, although depressed in level. The DV extent of the transcription factor Lmx1a was also significantly expanded throughout most of the Foxa2 progenitor domain. Lmx1a levels at the midline were consistently reduced, and in lateral regions were at even lower and graded levels (Figure 5A–F, Figure S3C–D, and data not shown). This result is consistent with ectopic Wnt signaling in the midbrain, in which Lmx1a is initially induced but ultimately attenuated [18]. Alternatively, progenitors might compensate for the overexpression of Lmx1b by downregulating the closely related Lmx1a.

To determine whether these domain changes were merely a consequence of overall increase in midbrain size, we measured the DV extent of the Lmx1a progenitor domain, the Foxa2 progenitor domain, and the length of the third ventricle (3V). By normalizing the DV extent of Lmx1a to the DV extent of Foxa2, and the DV extent of Foxa2 to the length of the third ventricle we determined that these domains are specifically expanded, rather than a consequence of the general increase in midbrain size (Figure 5A–I; Foxa2/3V shows a 20% increase, n = 3, control mean = 0.29±0.006, mutant mean = 0.35±0.02; p-value = .009). Moreover, the entirety of the Nkx6.1 progenitor domain is expanded, but intermingled with Lmx1a progenitors in these mutants (Figure 5J–K). As reported in previous studies [27], [47], [48] Lmx1a/b expansion led to repression of Nkx6.1, but likely because Lmx1a levels in lateral regions were not as robust as at the midline, this repression was only partial (i.e. several progenitors coexpressed Lmx1a and Nkx6.1). As a result, ectopic Lmx1a+ neurons, which appear to have increased Lmx1a levels after exiting the ventricular zone, were seen emanating from lateral aspects of the Foxa2 domain in addition to Nkx6.1+ neurons (Figure 5J–K). Further, we observed an increase in the DV extent of *Shh*, but a slight reduction in levels (Figure 5L–M). This result is consistent with an increase in Wnt signaling (see Figure 4) [17].

The early expansion of the Lmx1a/Foxa2 domain ultimately led to severe disruptions in ventral midbrain neuron types at later stages. Quantification of 13.5 dpc sections showed a 194% increase in Lmx1a+ cells and an 80% increase in TH+ mature mDAs. Brn3a+ red nucleus neurons were increased by 60%, likely because the overall Nkx6.1 progenitor domain was also expanded despite partial repression by Lmx1a. In contrast, a dramatic loss (90%) of Islet+ oculomotor neurons was observed (Figure 5N–U).

At 14.5 dpc, in addition to ectopic TH+ neurons in lateral regions, we observed a decrease in TH+ neurons at the midline, particularly in rostral sections (Figure S3I–R). Many cells at the midline appear to be stalled at the Nurr1+TH− state. This is likely because excess Lmx1b leads to increased *Wnt1*/Wnt signaling, too much of which is detrimental for normal dopamine neuron differentiation [20]. Alternatively, since a small Nurr1+/TH− population does exist in the wildtype postnatal midbrain, the increase in this population could indicate a change in fate. These data collectively demonstrate that failure to restrict Lmx1b during early embryogenesis drastically increases the third ventricle size and alters patterning of the midbrain. Further, excessive Lmx1b within the dopamine progenitor domain (see Figure S3E–F) is detrimental for normal dopamine differentiation, suggesting a need for careful modulation of its expression level.

Next, to determine whether Lmx1b was required for normal midbrain development, we examined *En1::Cre;Lmx1bcKO* embryos. Such embryos generated a complementary phenotype to the *En1::Cre;Lmx1bOE* in that the length of the third ventricle and midbrain size were reduced (Figure S4K–L). This reduction is at least in part due to apoptosis, as activated Caspase-3+ cells were increased, particularly in lateral midbrain regions (Figure S4A–B). Additionally, in *En1::Cre;Lmx1bcKO* mutants some *Otx2+* cells were detected across the isthmic boundary in the hindbrain (Figure S4M–N). *Fgf8* was drastically reduced in the isthmic region (Figure S4O–P).

**Figure 6:**
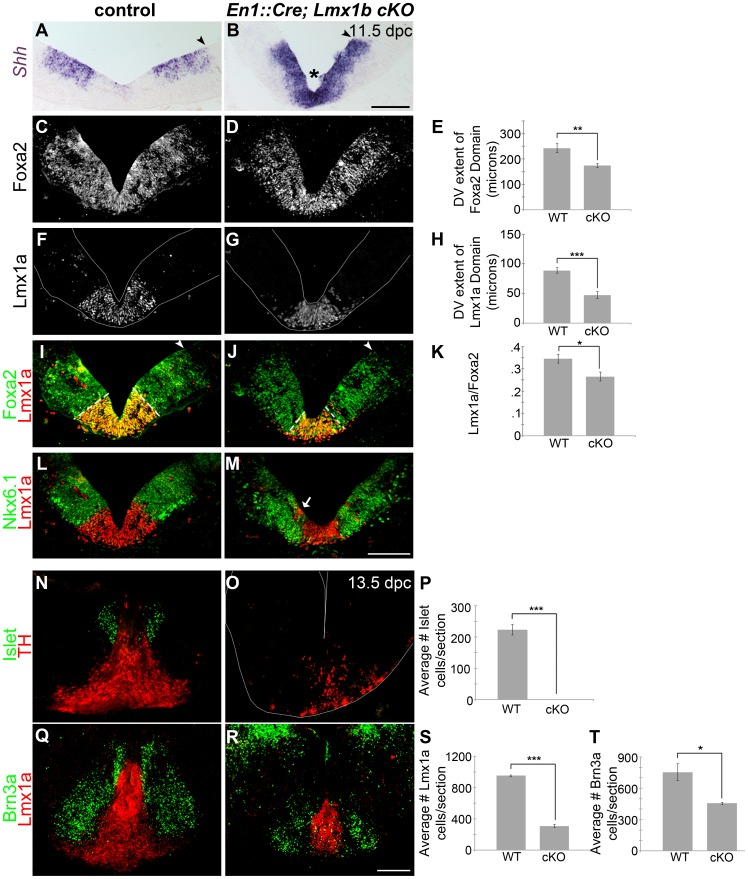
Loss of *Lmx1b* alters the size of the FP, the mDA progenitor domain, and ventral midbrain patterning. (A–B) In 11.5 dpc *En1::Cre,Lmx1bcKO* embryos, the cumulative *Shh* domain is reduced along the dorsal-ventral axis (black arrowheads), but *Shh* is robustly maintained at the midline (asterisk), indicative of reduced Wnt signaling. (C–M) Immunolabeling revealed a reduction in the size of the Foxa2 and Lmx1a progenitor domains. The closed white arrowheads indicate the dorsal border of Foxa2 expression, graphically represented in E (n = 3; control mean = 242.84±17.51 µM, mutant mean = 174.10±7.10 µM; p-value = 0.002). The dotted white lines indicate the dorsal border of Lmx1a expression, graphically represented in H (n = 3; control mean = 88.69±4.69 µM, mutant mean = 47.42±5.66 µM; p-value = 0.0004) and as a normalized ratio in K (p-value = 0.05). The main Lmx1a domain is reduced in size, but a few Lmx1a+ cells were observed within Nkx6.1+ territory (white arrow). The Nkx6.1 domain encroaches on the outer edges of the normal Lmx1a domain, and is only modestly reduced in size. (N–T) In 13.5 dpc *En1::Cre;Lmx1bcKO* embryos we found a nearly complete loss of Islet+ oculomotor neurons (n = 3; control mean = 223.11±16.42, mutant mean = 0.33±0.33; p-value = .0002), a 39% decrease in Brn3a+ red nucleus neurons (n = 3; control mean = 752.22±82.42, mutant mean = 455.78±10.56; p-value = .02), and a 68% decrease in Lmx1a+ mDA lineage cells (n = 3; control mean = 951.33±10.63, mutant mean = 308.89±18.99; p-value = 7.84E = 06). Sections in F, G, and O were outlined to accentuate tissue. Scale bars represent 100 µM.

Upon examination of the ventral midbrain of *En1::Cre;Lmx1bcKO* embryos, we observed that the DV extent of the *Shh+* FP domain was reduced (Figure 6A–B). However, *Shh* appeared to be maintained rather than downregulated at the midline in mutant embryos, in accordance with the fact that *Wnt1*/Wnt signaling is reduced (see Figure 4N and P) [17]. Further, the DV extent of the Foxa2+ FP was reduced (Figure 6C–E, Figure S4C–D). After normalizing the DV extent of Foxa2 to the 3V, this reduction was found to be proportionate to the reduction in midbrain size (Foxa2/3V shows a 12% decrease, n = 3, control mean = 0.31±0.01, mutant mean = 0.28±0.01; p-value = .066). Within this domain, the Lmx1a+ dopamine progenitor domain was selectively reduced (Figure 6F–K, Figure S4E–H), similar to mice with defects in *Wnt1*/Wnt signaling [15], [17]. Likely, however, due to some residual *Wnt1*/Wnt signaling in *En1::Cre;Lmx1bcKO*, the morphological changes seen in the *Wnt1* knockout and *Shh::Cre;Ctnnb1cKO* are not observed, and the distribution of remnant Lmx1a+ cells is different. Moreover, outside the main mDA progenitor domain, some Lmx1a+ cells were stranded within Nkx6.1+ territory, likely reflecting a failed attempt to establish a broader dopamine progenitor domain in this mutant. The Nkx6.1 territory was also reduced albeit not as drastically (22% reduction, n = 3, p-value = 0.06)(Figure 6L–M).

The early reduction of the Lmx1a/Foxa2 domain ultimately led to the diminution of many ventral midbrain neuron types at later stages. TH, a mature mDA marker, was drastically reduced, and quantification of 13.5 dpc sections revealed a 68% reduction of Lmx1a+ nascent mDAs. Brn3a+ red nucleus neurons showed a milder 39% reduction, likely reflective of a slight decrease in Nkx6.1+ progenitors. Only a few Islet+ oculomotor neurons were detected at 9.5 dpc, and these were virtually undetectable by 13.5 dpc (Figure 6N–T, Figure S4I–J). Altogether these data suggest that in the early embryo, Lmx1b is a key determinant of midbrain size, isthmic integrity, FP size, mDA progenitor domain size, and ventral neuron numbers.

### microRNAs are necessary for proper allocation of FP progenitors

**Figure 7:**
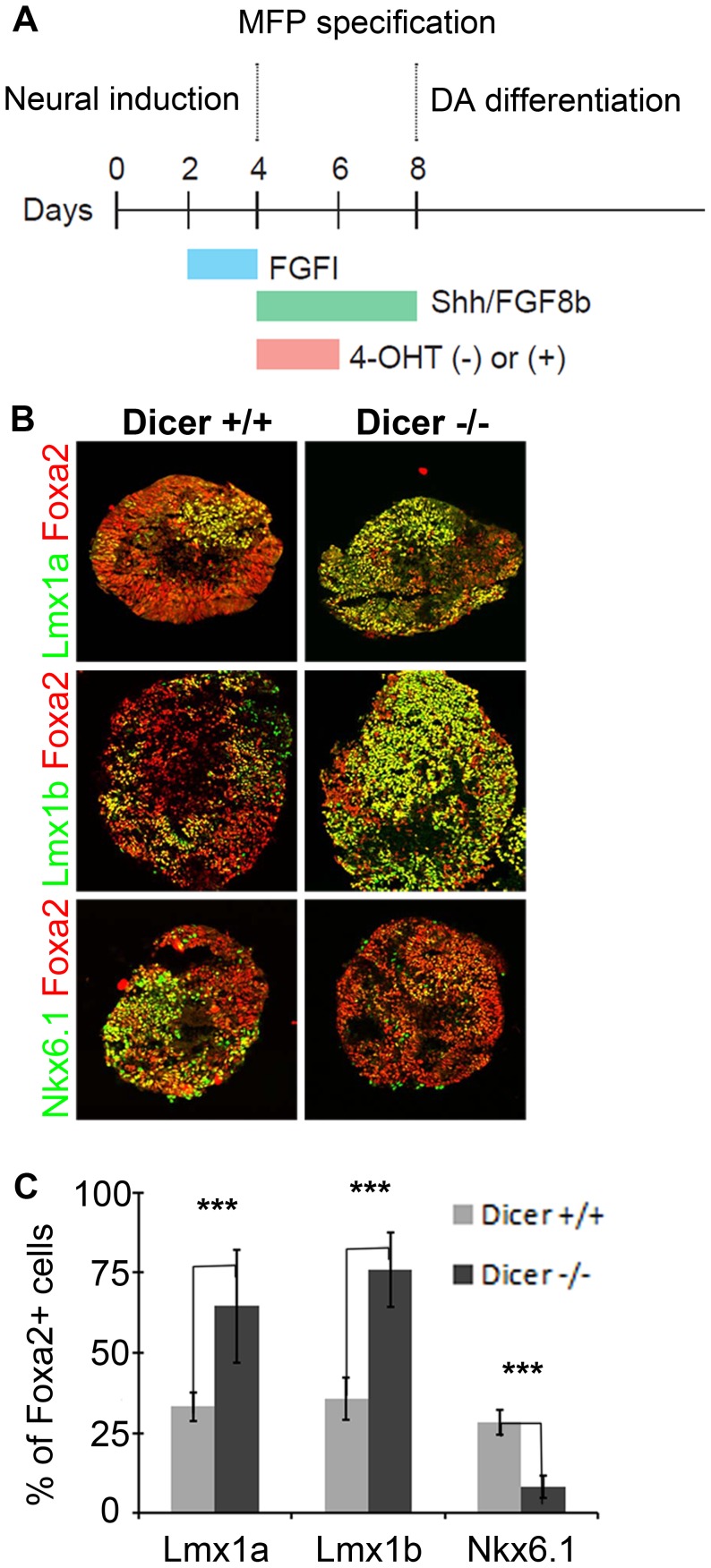
Embryoid bodies lacking *Dicer1* display FP progenitor allocation changes. (A) Protocol used for mDA differentiation from embryoid body aggregates and conditional removal of *Dicer1*. (B) In the absence of *Dicer1*, Lmx1a/b+ mDA progenitors are increased at the expense of Nkx6.1+ progenitors. (C) Unbiased Metamorph analysis to quantify marker expression in Foxa2+ progenitors (n = 15 embryoid bodies; Lmx1a control mean = 33.19±4.49, Lmx1a mutant mean = 64.55±17.46; Lmx1a p-value = 4.94E-09/Lmx1b control mean = 35.71±6.61, Lmx1b mutant mean = 75.94±11.65; Lmx1b p-value = 1.55E-10/Nkx6.1 control mean = 28.32±3.73, Nkx6.1 mutant mean = 8.28±3.36; Nkx6.1 p-value = 2.02E-10).

Since the proper dosage of transcription factors in the FP is imperative for determining progenitor allocation between the Lmx and Nkx6.1 domains, we next tested whether microRNAs played a role in regulating this process. To do this, we used an ESC line that harbored a *CAG::CreER^T2^* construct and *Dicer1* ^floxed/floxed^ alleles, such that Dicer1, the key microRNA processing enzyme, could be deleted upon 4-hydroxy tamoxifen (4OHT) administration [5]. We next developed an optimized protocol to derive dopamine progenitors from embryoid body aggregates (Figure 7A) [49]. Remarkably, in controls, we were able to achieve conditions that mimic the *in vivo* midbrain FP, wherein most progenitors were Foxa2+, and of these roughly equal numbers were Lmx1a/b+ and Nkx6.1+. We quantified the proportion of Foxa2+ cells that were Lmx1a+, Lmx1b+ and Nkx6.1+ in controls and mutants. In both controls and mutants, large numbers of Foxa2+ cells were observed. In controls, the proportion of cells that were Lmx1a+, Lmx1b+, or Nkx6.1+ was roughly equivalent. In 4OHT treated cultures, however, the proportion of Lmx1a/b+ cells was drastically increased, while the proportion of Nkx6.1+ cells was decreased (Figure 7B–C). These data suggest that microRNAs are involved in progenitor cell allocation between the Lmx1a/b+ and Nkx6.1+ domain in ESC derived cultures, and under these conditions, de-repression of target genes through the loss of microRNAs expands mDA progenitors.

### Early *miR135a2* overexpression reduces *Lmx1b/Wnt1*/Wnt signaling and phenocopies loss of Lmx1b

**Figure 8:**
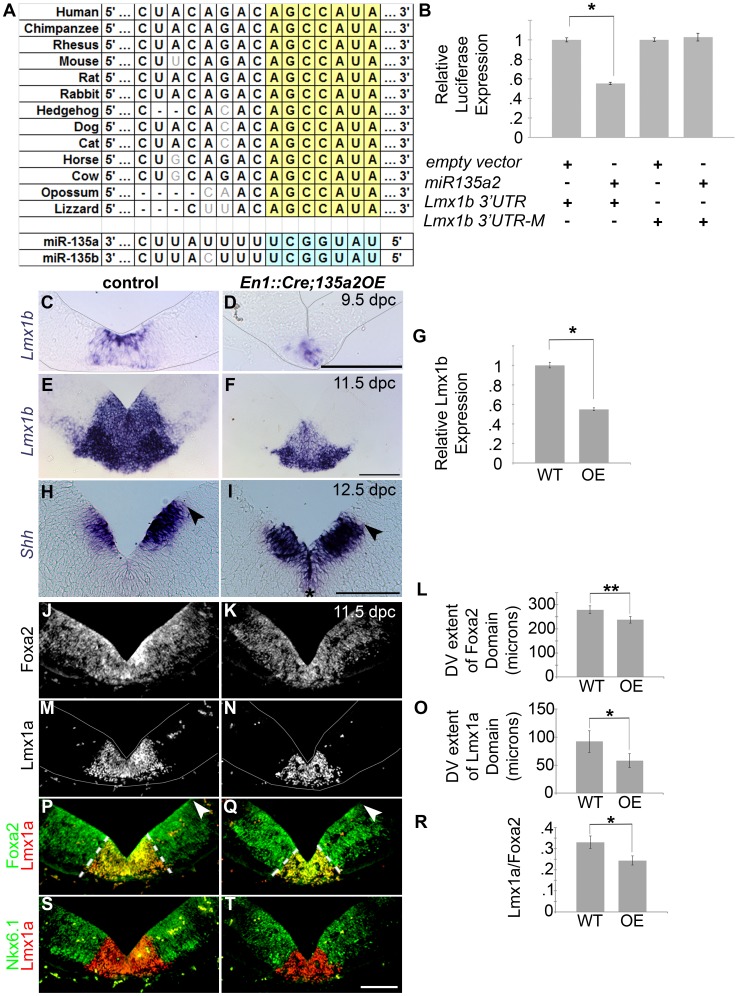
Increasing *miR135a2* levels reduces mDA progenitor domain size and alters allocation of mDA progenitors. (A) *miR135a/b* binding sites are conserved in the *Lmx1b 3′UTR*. (B) Transient transfection in HEK293 cells shows that *miR135a2* is sufficient to repress a construct containing a fragment of the *Lmx1b 3′UTR* (n = 16; empty vector mean = 1±.14, *miR135a2* mean = .63±.06; p-value = .02), but not a construct (*Lmx1b-M2*) harboring a mutation in the *miR135* binding site (n = 16; empty vector mean = 1±.11, *miR135a2* mean = 1.02±.12; p-value = .91). (C–F) *In situ* hybridization in *En1::Cre;135a2OE* at 9.5 dpc and 11.5 dpc shows that *Lmx1b* is not abolished. Rather, the *Lmx1b* domain size is reduced. In the remnant *Lmx1b* domain, *Lmx1b* levels appear slightly reduced. These data suggest that the outer edges of the *Lmx1b* domain are more vulnerable to microRNA overexpression. (G) Quantitative Real Time RT-PCR further confirms that *miR135a2* overexpression is sufficient to repress *Lmx1b in vivo*. In 8–14 somite stage *En1::Cre;135a2OE* embryos *Lmx1b mRNA* is reduced by 45% (n = 6; control mean = 1±0.03, mutant mean = 0.55±0.01; p-value = .03) (H–I) At 12.5 dpc the dorsal-ventral extent of the *Shh* domain was reduced in size (black arrowheads indicate dorsal border of expression) and the level of *Shh* expression was slightly maintained at the midline of many embryos analyzed (black asterisk). (J–T) Immunolabeling revealed a reduction in the size of the Foxa2 and Lmx1a progenitor domains. The closed white arrowheads indicate the dorsal border of Foxa2 expression, graphically represented in L (n = 4; control mean = 278.23±16.12 µM, mutant mean = 236.83±13.77 µM; p-value = 0.01). The dotted white lines indicate the dorsal border of Lmx1a expression, graphically represented in O (n = 4; control mean = 92.06±19.39 µM, mutant mean = 57.97±12.24 µM; p-value = 0.03) and as a normalized ratio in R (p-value = 0.05). In panels M–N, P–Q, and S–T some background staining was found in blood vessels outside the mDA domain. The Nkx6.1 domain encroaches on the outer edges of the normal Lmx1a domain, and is minimally altered in size. Sections in C–D and M–N were outlined to accentuate tissues. Scale bars represent 100 µM.

We sought to determine if *miR135a2* is sufficient to repress *Lmx1b*, and can thereby modulate midbrain development. To test this hypothesis, we first performed a luciferase assay in HEK293 cells, and found that *miR135a2* was able to repress a construct harboring a fragment of the *Lmx1b 3′UTR*, but not constructs harboring mutations within the evolutionarily conserved *miR135a2* binding site of the *Lmx1b 3′UTR* (Figure 8A–B and data not shown).

Next, we generated transgenic mice that conditionally express a *mmu-miR-135a-2* precursor under control of *CAG* elements (*CAG-loxP-STOPr-loxP-miR135a2-IRESeGFP*; see Materials and Methods for description); in dissected 8.5 dpc midbrain, *miR135a* was detected in controls, and was approximately 3 fold increased in *En1::Cre;135a2OE* mutants (Figure S5D), although eGFP activity was not detectable (see Materials and Methods). *In situ* hybridizations showed a reduction in the domain size and levels of *Lmx1b* (Figure 8C–F), which was confirmed by qRT-PCR (Figure 8G) and indicated that *miR135a2* is sufficient to repress *Lmx1b in vivo*. *Otx2*, which harbors a binding site for the closely related *miR135b*, is also decreased by both *in situ* hybridization and q-RT-PCR (Figure S6I–K); however, it remains to be determined whether *Otx2* levels are decreased due to direct microRNA mediated repression, or in response to reduced *Wnt1*/beta-catenin signaling, or both (Figure S7).

We reasoned that overexpressing *miR135a2* should, at least in part, phenocopy *Lmx1b*-deficient embryos. Indeed, in *En1::Cre;135a2OE* embryos obtained from three separate transgenic lines (Figure S5A–C, and data not shown), we observed an overall reduction in midbrain size (Figure S6A–B and J–K), albeit not as drastic as *En1::Cre;Lmx1bcKOs*. In 9.5 dpc *En1::Cre;135a2OE* embryos, we detected an increase in the apoptotic marker activated Caspase-3 (Figure S6A–B). Large numbers of apoptotic cells were observed in lateral regions of the midbrain, whereas few were detected in the ventral midbrain. In 10.5 dpc and 11.5 dpc embryos, apoptosis is markedly reduced relative to 9.5 dpc embryos (data not shown). Increased apoptosis, particularly at 9.5 dpc, could at least in part underlie the size reduction of the midbrain.

We next examined the progenitor domains in the ventral midbrain of *En1::Cre;135a2OE*. The DV extent of the *Shh* domain was decreased, and in many mutants analyzed (n = 6/10), *Shh* was not as robustly downregulated at the midline as in controls (Figure 8H–I). In one particularly severe mutant, *Shh* was maintained at the midline in a manner identical to *En1::Cre;Lmx1bcKO* embryos (Figure S6P–Q). The DV extent of the Foxa2 domain was also decreased (Figure 8J–L and S6C–D). After normalizing the DV extent of Foxa2 to the 3V, this reduction was found to be proportionate to the reduction in midbrain size (Foxa2/3V revealed an 8% decrease, n = 4, control mean = 0.28±0.02, mutant mean = 0.24±0.01; p-value = .28). Further analysis of the progenitor populations within the Foxa2 domain showed disproportionate changes. We observed a dramatic reduction in the dimensions of the Lmx1a/b+ domain, both in width (i.e. dorsal – ventral dimension) and thickness (i.e. ventricular – pial dimension). This reduction in the Lmx1a domain was not solely due to a reduction in FP size. When measured relative to Foxa2, the Lmx1a domain still appeared selectively reduced, as the width of the neighboring Nkx6.1+ domain was largely unaffected (Figure 8M–T and S6E–H).

**Figure 9:**
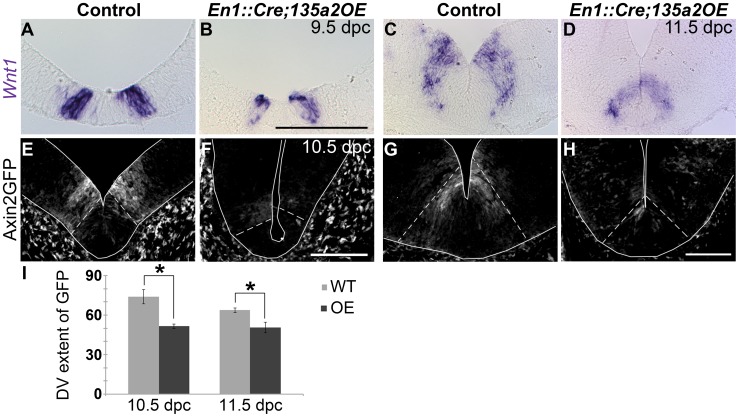
Increasing *miR135a2* levels diminishes *Wnt1* and Wnt signaling. (A–D) *In situ* hybridization in 9.5 dpc and 11.5 dpc *En1::Cre;135a2OE* embryos revealed a reduction in the size of the *Wnt1* expression domain. (E–H) At 10.5 dpc, some *Axin2::d2eGFP* transgene activity was observed to be inside the Lmx1a domain (dotted white lines), but d2eGFP was predominantly detected outside of this domain. At 11.5 dpc d2eGFP only extend a few cell diameters outside of the Lmx1a domain (dotted white lines). In most mutant sections at both ages, the intensity of GFP labeling was diminished at all anterior-posterior levels of the ventral midbrain. (I) Quantification of the distance that GFP labeling was observed along the dorsal-ventral axis showed a 30% reduction in 10.5 dpc mutants (n = 3; control mean = 73.99±5.34 µM, mutant mean = 51.50±1.77 µM; p-value = 0.02) and a 21% reduction in 11.5 dpc mutants (n = 3; control mean = 63.68±1.72 µM, mutant mean = 50.44±4.03 µM; p-value = 0.04). (GFP labeled sections were outlined to accentuate tissue). Scale bars represent 100 µM.

Since Lmx1b is upstream of *Wnt1*/Wnt signaling, and *Wnt1*/Wnt signaling is important in mDA progenitor specification, we examined alterations in *Wnt1*/Wnt signaling in *En1::Cre;135a2OE* embryos. From 9.5–11.5 dpc, the levels of *Wnt1* and the size of the *Wnt1* expression domain, which is included within the *Lmx1b* domain, appeared to be reduced (Figure 9A–D). Although the reduction was not as drastic as in the *En1::Cre;Lmx1bcKO*, and is unlikely to singularly explain this phenotype, these results suggested that the amount of available Wnt1 ligand in the ventral midbrain was restricted.

We next examined ongoing Wnt signaling in the ventral midbrain using the *Axin2::d2eGFP* allele described above, which provides a transcriptional readout of robust canonical Wnt signaling [43], [44]. In control 10.5 and 11.5 dpc embryos harboring an *Axin2::d2eGFP* transgene, d2eGFP is observed in the ventral midbrain in two stripes adjacent to the midline. At 10.5 dpc, GFP labeling is predominantly observed outside of the Lmx1a domain, although some is detected within the domain. At 11.5 dpc, GFP labeling still exceeds the Lmx1a domain by several cell diameters along the DV axis. In comparison to controls, *En1::Cre;135a2OE,Axin2::d2eGFP* mutants showed diminished GFP labeling intensity within the ventral midbrain, which was nearly extinguished in particularly severe embryos (Figure 9E–H). Further, consistent with the reduction in Wnt1 ligand, the total range of GFP labeled cells in *En1::Cre;135a2OE*,*Axin2::d2eGFP* embryos was reduced along the DV axis (Figure 9I). Little to no reduction of GFP was observed in the dorsal midbrain (data not shown). Although not as severe as the *En1::Cre;Lmx1bcKO*, reduced canonical Wnt signaling, which is critical for early activation and maintenance of Lmx1a [17], [46], could in part, explain the diminished mDA progenitor domain. At 11.5 dpc, reduced Wnt signaling is likely to be important for aspects of neurogenesis rather than the establishment of the mDA progenitor domain.

As *Lmx1b* and *Wnt1* are also present in the isthmus, we examined this region in *En1::Cre;135a2OE* embryos. Analysis of the midbrain/hindbrain junction revealed differences between the *En1::Cre;Lmx1bcKO* and *En1::Cre;135a2OE* mutants. While Lmx1b, *Wnt1*, and *Fgf8* were modestly, but consistently, narrower in *En1::Cre;135a2OE* mutants, the isthmic boundary, as determined by *Otx2*, was unchanged (Figure S8A–H). In contrast, in the isthmus of *En1::Cre;Lmx1bcKO* embryos, both *Wnt1* (Figure 4T) and *Fgf8* (Figure S4P) were drastically reduced, and Otx2+ cells were found in the rostral hindbrain (Figure S4N).

**Figure 10:**
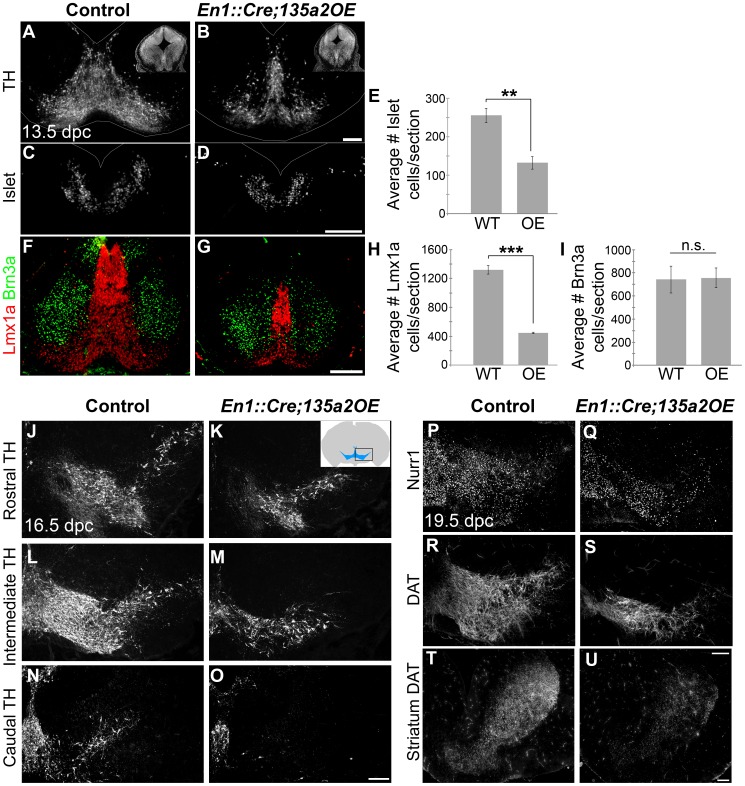
Reduced numbers of mDAs in *En1::Cre;135a2OE* embryos. (A–B) Analysis of 13.5 dpc *En1::Cre;135a2OE* embryos revealed a significant loss of TH+ mDA neurons (insets are DAPI images depicting the reduction in midbrain size). (C–E) Quantification of Islet+ cells in control and mutant embryos revealed a 48% reduction (n = 3; control mean = 255.46±18.59, mutant mean = 132.08±16.18; p-value = .008). (F–I) Quantification of Lmx1a+ cells in control and mutant embryos revealed a 66% reduction (n = 3; control mean = 1319.67±59.31, mutant mean = 447.5±8.39; p-value = .0001). In contrast, Brn3a+ red nucleus neuron numbers are not drastically altered (n = 3; control mean = 742.22±114.93, mutant mean = 757.33±83.82; p-value = .92). (J–O) TH+ neurons are reduced throughout the anterior posterior axis in 16.5 dpc mutant embryos. (P–S) 19.5 dpc mutant embryos reveal that although there are significantly fewer dopamine neurons, they do express normal mDA markers including Nurr1 and DAT. (T–U) DAT immunoreactivity in the striatum is markedly reduced. Sections in A–D were outlined to accentuate tissue. Scale bars represent 100 µM.

We next examined later stage embryos to determine the consequences of early reduction of the mDA progenitor population. In 13.5 dpc *En1::Cre;135a2OE* embryos we observed a drastic decrease of TH+ mDAs. Quantification revealed a 66% reduction in Lmx1a+ nascent mDAs, as well as a 48% reduction in Islet+ oculomotor neurons. In contrast, Brn3a+ neuron numbers in the red nucleus [24], were not altered (Figure 10A–I), likely reflective of the largely unaffected Nkx6.1+ progenitor domain. Moreover, several Brn3a+ cells were observed at the midline in the dopaminergic field (Figure 10F–G and Figure S9A–D), which is consistent with a reduction of the Lmx1b+ mDA progenitor domain [50], [51]. Further analysis at 16.5 dpc confirmed a drastic reduction of TH+ dopamine neurons throughout the anterior-posterior axis (Figure 10J–O). At 19.5 dpc we observed that the remnant neurons express mature mDA markers including Nurr1 and DAT (Figure 10P–S); this finding is different from reported *En1::Cre;Lmx1bcKO* studies [52] likely because in *En1::Cre;135a2OE* mutants, *Lmx1b* is reduced but not abolished. Consistent with the reduction of dopamine neuron numbers, a concomitant decrease of mDA projections to the striatum was observed (Figure 10T–U).

**Figure 11:**
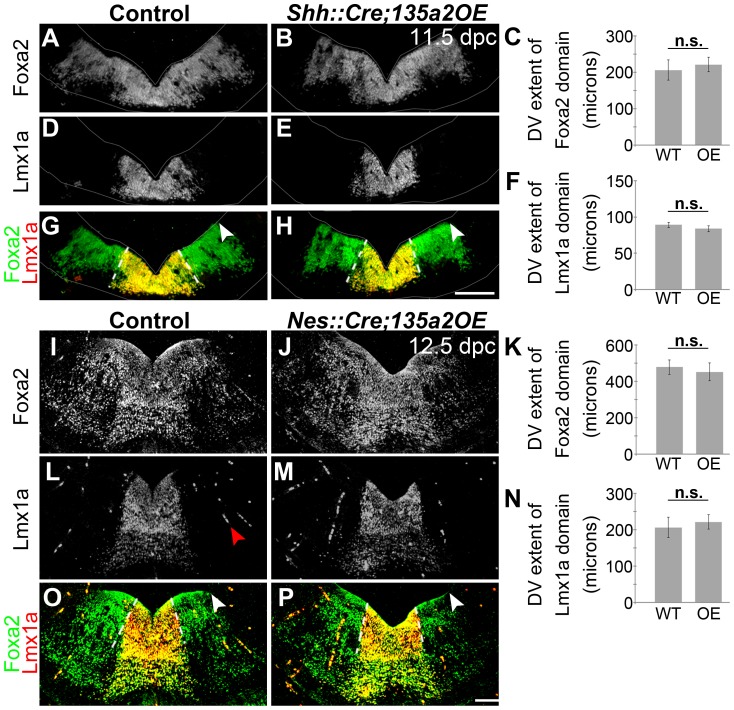
Increasing *miR135a2* levels after an early critical window has no effect on mDA progenitor domain size. (A–H) Immunolabeling of 11.5 dpc *Shh::Cre;135a2OE* embryos, in which recombination begins at the ventral midline at ∼8.5 dpc, revealed no change in the dorsal-ventral extent of either the Foxa2 or Lmx1a progenitor domains. The closed white arrowheads indicate the dorsal border of Foxa2 expression, graphically represented in C (n = 3; control mean = 229.34±6.51 µM, mutant mean = 221.17±10.68 µM; p-value = 0.55). The dotted white lines indicate the dorsal border of Lmx1a expression, graphically represented in F (n = 3; control mean = 89.09±3.49 µM, mutant mean = 83.72±4.13 µM; p-value = 0.38). The sections were outlined to accentuate tissue. (I–P) Similarly, analysis of 12.5 dpc *Nes::Cre;135a2OE*, in which recombination occurs throughout the region at ∼10.5 dpc, revealed no change in the dorsal-ventral extent of either the Foxa2 or Lmx1a progenitor domains. The closed white arrowheads indicate the dorsal border of Foxa2 expression, graphically represented in K (n = 3; control mean = 478.03±39.89 µM, mutant mean = 451.67±48.67 µM; p-value = 0.41). The dotted white lines indicate the dorsal border of Lmx1a expression, graphically represented in N (n = 3; control mean = 205.64±27.89 µM, mutant mean = 221.29±19.84 µM; p-value = 0.39). The red arrowhead in L points to background staining found in blood vessels outside the mDA domain. Scale bars represent 100 µM.

Next, we reasoned that because deletion of *Lmx1b* with a slightly later and more specific *Shh::Cre* driver reveals no changes in the size of the mDA domain [51], if *miR135a2* is indeed acting through the Lmx1b/Wnt axis, then using *Shh::Cre* to overexpress *miR135a2* should have no effect on mDA domain size. To determine whether *miR135a2*, could elicit mDA progenitor domain changes when activated at later stages (i.e. after the early restriction to the RP, FP, and IsO) we used both *Shh::Cre* and *Nestin (Nes)::Cre* recombination. When *miR135a2* overexpression was initiated at ∼8.5–9.5 dpc with *Shh::Cre* or ∼10.5 dpc with *Nes::Cre*, the DV extent of the Foxa2 and Lmx1a domains were unchanged (Figure 11).

To interpret these results, we carefully considered the spatiotemporal expression of the different Cre drivers. *En1* expression is initiated at the 2 somite stage and encompasses the prospective midbrain and rhombomere 1 regions, inclusive of the IsO, by the 4–6 somite stage (8.0 dpc) [53], [54]. In contrast, *Shh* is initiated slightly later at the 8-somite stage (8.5 dpc) [55], specifically at the ventral midline. At this early stage, however, *Shh* expression is confined to a small group of medially located progenitors, which does not encompass the entire prospective mDA progenitor domain [22], [28]. Subsequently, between 8.75 and 9.5 dpc, *Shh* expression flares out laterally, and encompasses the entire mDA domain [22]. If one considers the recombination of the entire prospective mDA progenitor domain, there is a significant time difference between *En1::Cre* and *Shh::Cre* recombination. *Nestin::Cre* is active throughout this region, including the IsO, between ∼10.5–11.5 dpc [56]. Considering these recombination kinetics, our results indicate that increased *miR135a2* levels are sufficient to restrict the mDA progenitor domain size only during an early critical window. Since Lmx1b is broadly expressed at the ventral midline during this time window [27], we posit that *miR135a2* expression aids in the DV restriction of Lmx1b and the refinement of the mDA progenitor domain; within the mDA domain, the outer edges appear more sensitive to increased *miR135a2* levels. An alternative interpretation of these results is that the spatial differences in recombination between *En1::Cre* and *Shh::Cre*, rather than solely the timing differences, lead to the differing effects on the mDA progenitor domain. Particularly, since *En1::Cre* recombines the entire midbrain-rhombomere 1 region, while *Shh::Cre* does not, it is possible in *En1::Cre;135a2OE* repression of the Lmx1b/Wnt1 axis results in a partial breakdown of the Wnt-Fgf loop [18], [57]. Thus, early defects in IsO activity cannot be ruled out, and could also contribute to the phenotype observed in *En1::Cre;135a2OE*.

By eight criteria: total midbrain size, apoptosis, *Wnt1*/Wnt signaling, DV extent of the FP (Foxa2/*Shh* domain size), mDA progenitor domain size, mDA numbers, oculomotor neuron numbers, and temporal sensitivity, the *miR135a2OEs*, at least partially resemble the *Lmx1bcKOs* (Figure S10). In several criteria tested, the *En1::Cre;Lmx1bcKO* is more affected than the *En1::Cre;135a2OE*, likely because *Lmx1b* levels are not completely abolished in the latter. Together with the *in vitro* luciferase assays, our data suggests that the *En1::Cre;135a2OE* phenotype is at least in part dependent on *Lmx1b* repression in the early embryo. Further, the *En1::Cre;Lmx1bcKO* and the *En1::Cre;135a2OE* phenotypes are similar because they may both, in part, be due to net deficits in Wnt signaling. In the *En1::Cre;Lmx1bcKO*, reductions in Wnt signaling are likely due to a massive deficit of Wnt1 ligand (Figure 4N). In the *En1::Cre;135a2OE*, reductions in Wnt signaling are likely due to modest reductions in several targets including multiple levels of the Wnt pathway (Figure 9, Figure S7, and Table 1), altogether resulting in a significant net Wnt signaling deficit.

## Discussion

**Figure 12:**
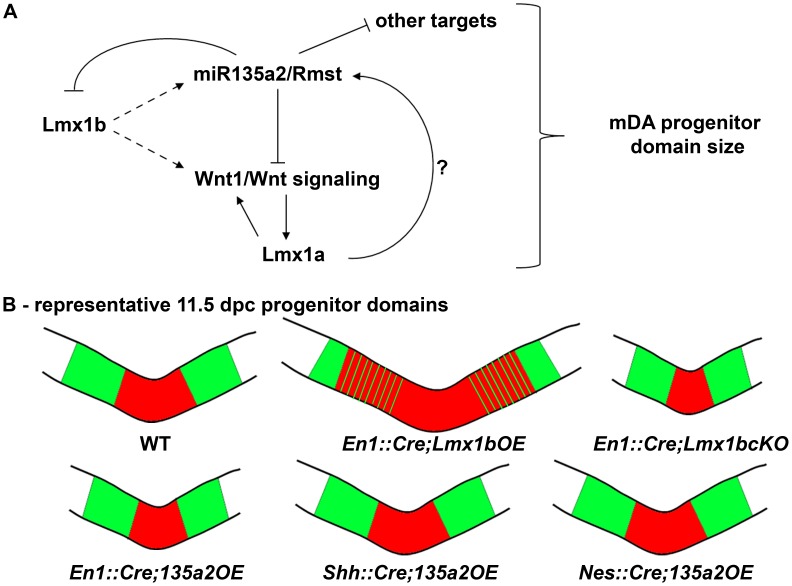
Opposing effects of *Lmx1b* and *miR135a2* on dopamine progenitor allocation. (A) Lmx1b, either directly or indirectly, drives the expression of *miR135a2/Rmst*, which in turn can negatively modulate the levels of *Lmx1b*, *Wnt1*/Wnt signaling, and other key factors. Together, these factors are critical for net *Wnt1*/Wnt signaling, which could influence mDA progenitor patterning and expansion, in part by inducing Lmx1a. Lmx1a also likely has the ability to drive *miR135a2/Rmst* expression, although this remains to be demonstrated. (B) Cartoon of the midbrain FP region. A balance of *Lmx1b* and *miR135a2* determines mDA progenitor allocation within the FP. *Lmx1b* maintenance (*En1::Cre;Lmx1bOE*) results in an increased mDA progenitor domain, which encroaches on the Nkx6.1 domain. Conversely, early loss of *Lmx1b* (*En1::Cre;Lmx1bcKO*) or increasing *miR135a2* levels (*En1::Cre;135a2OE*) results in a disproportionately decreased mDA progenitor pool. The outer edges of the mDA progenitor pool are most vulnerable to changes in Lmx1b, and are lost in both these mutants. Later *miR135a2* overexpression (*Shh::Cre;135a2OE* and *Nes::Cre;135a2OE*) results in no change in the DV extent of the mDA progenitor domain. The microRNA, thus acts as a rheostat to set the boundary between the Lmx (red) and Nkx6.1 (green) domains only during an early critical window.

In this study we have identified a novel transcription factor/microRNA negative feedback loop that critically impacts *Wnt1*/Wnt signaling and midbrain development. Feedback circuitry inherently possesses a powerful buffering capacity, such that fluctuations in gene expression can be stabilized and protein expression in dynamically changing environments can be fine-tuned [58]. In this case, widespread *Lmx1b/Wnt1*/Wnt signaling must be sharply restricted over a short period (between 8.0 and 9.5 dpc), as maintenance of *Lmx1b/Wnt1*/Wnt signaling leads to several unwanted consequences. Here, we provide evidence that *Lmx1b* is upstream of *miR135a2/Rmst*, and that *miR135a2* represses *Lmx1b/Wnt1*/Wnt signaling (Figure 12A). Thus, in the early midbrain, these factors may determine mDA progenitor allocation and midbrain size.

Our data indicate that microRNAs play a critical role in determining the size of the mDA progenitor pool. We found that loss of the key microRNA processing enzyme, Dicer1, in embryoid body aggregates skews the proportion of Foxa2+ progenitors in favor of an Lmx1a/b+ mDA fate over an Nkx6.1+ fate. Conversely, we found that early *miR135a2* overexpression *in vivo* led to a disproportionate reduction in the size of the Lmx1a/b+ mDA progenitor domain. We propose that *miR135a2* might influence net Lmx stoichiometry in two ways: first, by directly repressing *Lmx1b*, and second, by lowering *Wnt1*/Wnt signaling and therefore *Lmx1a* levels. In the early embryo, these alterations most prominently affect the outer edges of the Lmx domain, which mainly express Lmx1b, until Lmx1a is induced in this region [27]. Additionally, it is possible that lowering Wnt signaling alters proliferation, and that can in part account for the reduced mDA progenitor domain size.

We postulate that because of the reduction in *Lmx1b* and *Wnt1*/Wnt signaling in *En1::Cre;135a2OE* mutants, the Lmx1a+ domain fails to expand, ultimately resulting in a region where both Lmx1a and Lmx1b are extinguished and instead occupied by Nkx6.1. Supporting the notion that the boundaries are most vulnerable to changes in Lmx levels, even complete removal of *Lmx1b* in the *En1::Cre;Lmx1bcKO* results in the selective failure to establish the outer edges of the mDA progenitor pool. Taken together, our data indicate that the precise balance of *Lmx1b* and *miR135a2* at early time points in the embryonic midbrain determines the size of the dopamine progenitor domain (Figure 12B), and ultimately affects the organization and number of neurons found within different ventral midbrain populations.

Our data show that both increased or decreased *Lmx1b* expression in the early embryo results in reduced oculomotor neuron numbers. In the early midbrain, Lmx1b is transiently expressed in the oculomotor primordium [50]. Based on this, one possible result was that prolonged maintenance of Lmx1b would lead to increased oculomotor neurons in *En1::Cre;Lmx1bOE* mutants, but we found the opposite (Figure 5N–P). Thus, we conclude that *Lmx1b* downregulation is critical for production of normal oculomotor numbers. *miR135a2* may play a role in the timely regulation of Lmx1b in this context.

Precise and dynamic expression of the *Lmx1b-miR135a2* duo is also important for determining overall midbrain size. Failure to restrict *Lmx1b* leads to an enlargement of the third ventricle and morphogenetic abnormalities, while excess *miR135a2*-mediated repression or loss of *Lmx1b*, leads to a reduction in third ventricle and midbrain size. Since *Lmx1b* drives *Wnt1* expression, the phenotypes in *En1::Cre;Lmx1bOE*, *En1::Cre;Lmx1bcKO* and *En1::Cre;135a2OE* mutants can at least in part be attributed to alterations in early Wnt signaling. In accordance with this notion, *Wnt1*/Wnt signaling has been shown to determine the overall size of the midbrain by influencing cell survival and proliferation, [38], [54], [59]–[62]. The potency of *Wnt1*/Wnt signaling in midbrain development suggests that a method to control its stoichiometry is critical; we propose that the dose and spatiotemporal expression of *Wnt1*/Wnt signaling is, at least in part, determined by *Lmx1b* and *miR135a2*.

*miR135a2* is predicted to regulate a large set of target genes in addition to *Lmx1b*. *Lmx1a*, a related transcription factor, is not a predicted target by any algorithm we encountered. However, some of the genes in the predicted *miR135a2* target set, including *Wnt1, Wnt5a*, several molecules in the canonical Wnt signaling pathway (Figure 9, Figure S7, and Table 1), and *Msx1/2* (Anderegg, unpublished observations), may play a critical role in midbrain development and contribute to the observed phenotypes. Moreover, *Otx2*, a transcription factor also upstream and downstream of *Wnt1*/beta-catenin signaling [17], [29], [63], whose dosage determines the size of the mDA progenitor pool [63], is a predicted target of the closely related *miR135b* (Anderegg, unpublished observations). Since the seed is identical in these two microRNAs, it is possible that *Otx2* dosage is also controlled, in part, by *miR135a2*, although our luciferase assays could not detect a significant interaction (see Figure S7B).

It also remains possible that *miR135a2* acts on other signaling pathways important for midbrain development including TGF-beta/Bmp [64], [65], Shh [66], and Fgf [54], [67], either directly or indirectly through points of crosstalk. In fact, the mDA phenotype observed in *En1::Cre;135a2OE* mutants bears some resemblance to that of *En1::Cre;Fgfr* triple knockout mice [67]. However, parallel data obtained from *miR135a2* overexpression in the forebrain resulting in phenotypes that overlap Wnt mutants, supports the notion that defects in Wnt signaling contribute to the observed midbrain phenotype (Caronia-Brown, unpublished observations). Additionally, in colorectal cancers, *miR135a* targets the Wnt pathway, although in that context, with net positive effect [68]. By potentially targeting multiple levels of the Wnt pathway (see Table 1), including upstream transcription factors (*Lmx1b* and *Otx2*), ligands, positive and negative modifiers, and downstream targets it is likely that *miR135a2* confers precision to the stability of the overall early Wnt regulatory network responsible for midbrain size and dopamine progenitor specification. In this context, it is conceivable that the modest increase in *miR135a2* levels in *En1::Cre;135a2OE* modulates multiple Wnt pathway targets resulting in a net negative Wnt signal; in contrast, in *En1::Cre;Lmx1bcKO*, a net negative Wnt signal is primarily obtained by severe downregulation of *Wnt1*.

Our model is based on several lines of evidence derived from the mutants presented here. Our data suggest that *miR135a2* is sufficient to modulate canonical Wnt signaling and the mDA progenitor phenotype, but it does not address whether *miR135a2* is necessary for regulation of these phenotypes. Future loss of function studies are important to address this question and are currently underway. Since single microRNA knockouts often have subtle or no phenotypes, a double knockout of *miR135a2* and closely related *miR135b* will likely be required to unmask the functions of this microRNA family [69]. Given the importance of the Wnt pathway in disease and cancer, however, the gain of function experiments described here are an important advance, as they provide a novel modality for targeting this pathway.

The *Lmx1b*-*miR135a2* pair appears to be an integral component in the molecular circuitry governing establishment of mDA progenitors. Here we have demonstrated that this pair determines early aspects of mDA progenitor domain allocation, likely via direct effects on the ventral midbrain but possibly through IsO activity as well. However, after the size and spatial boundaries of the dopamine progenitor domain are firmly established, it is likely that *miR135a2* has a distinct, later role of tuning, and ultimately downregulating *Lmx1b*/*Wnt1* expression within the mDA progenitor pool (see Figure 3). This later role could be to tune optimal levels of Wnt signaling in the mDA domain, as well as aspects of neurogenesis. Thus, understanding this circuit may shed light on dopamine neuron numbers, and may therefore be relevant for understanding dopamine related disorders and susceptibilities to these conditions.

Generating large numbers of bona fide mDAs is a key goal of stem cell based approaches towards Parkinson's disease treatment. Previously, this process was limited by the inability to produce authentic mDAs that survived grafting. Recently this problem was overcome, using *Wnt1*/Wnt agonists to program hESCs towards a bona fide mDA fate [70], [71]. In this context, understanding agents that modulate *Wnt1*/Wnt signaling is of critical importance. It is conceivable, that just as iPS cells can now be programmed using microRNAs [72], mDAs will be derived from stem cells with the aid of a rationally designed cocktail of microRNAs and anti-microRNAs.

## Materials and Methods

### Ethics statement

All mouse work was done in accordance with Northwestern University's ACUC guidelines.

### MicroRNA array

Tissue was microdissected from the ventral midline and dorsal lateral midbrain of unfixed, coronally sectioned 11.5 dpc Swiss Webster embryos. Total RNA, including small RNAs, was extracted using the *mir*Vana kit (Ambion). The TaqMan Rodent MicroRNA A+B Cards Set v2.0 (4400239) was used to perform the array and validation of individual miRs was done with TaqMan PCR Assays for *miR135a* (ID 000460), *miR135b* (ID 002261), and normalized to *snoRNA202* (ID 001232)(Applied Biosystems)(see below).

### Quantitative Real Time RT-PCR (qRT-PCR)

For *miR135a* analysis in various mutant lines, tissue was dissected from littermates and snap frozen on liquid nitrogen. The meso-diencephalic region was dissected from older embryos (9.75 dpc *En1::Cre;Lmx1bOE*, 9.75 dpc *En1::Cre;Lmx1bcKO*, and 11.5 dpc *Nes::Cre;135a2OE*), while the whole head was used for younger embryos (8.75 dpc *En1::Cre;135a2OE*). Total RNA, including small RNAs, was extracted using the *mir*Vana kit (Ambion). The TaqMan PCR Assay for *miR135a* (ID 000460) was used and normalized to *snoRNA202* (ID 001232)(Applied Biosystems).

For transcription factor analysis, ∼8.5 dpc *En1::Cre;135a2OE* littermates were used. A cut was made just caudal to the future rhombomere 1, and the tissue from the head was snap frozen on liquid nitrogen. Total RNA was extracted using the *mir*Vana kit (Ambion) then the High Capacity cDNA Reverse Transcription Kit (Applied Biosystems) was used to generate cDNA. The TaqMan gene assay for *Lmx1b* (Mm00440209_m1) was used to analyze RNA from 8–14 somite embryos, and the TaqMan gene assay for *Otx2* (Mm00446859_m1) was used to analyze RNA from 13–23 somite embryos. Gene expression was normalized to *Gapdh* (Mm99999915_g1)(Applied Biosystems).

For all qRT-PCR experiments, the amount of total RNA per reaction was the same for each sample. The delta Ct method was used to calculate the relative fold changes in gene expression. All statistical values were calculated using a two-sample equal variance, two tailed, Student's t-Test.

### *Rmst* RT-PCR

Ventral midbrain tissue was dissected from 11.5 dpc Swiss Webster embryos and flash frozen on liquid nitrogen. Total RNA, including small RNAs, was extracted using the *mir*Vana kit (Ambion). A cDNA library was synthesized using SuperScript II Reverse Transcriptase (Invitrogen) and random hexamers. PCR was performed with the following primers:

exon 12 (F) GCTAGCTCGAGAGCCACGCTCTTTCCCAACAC

exon 15 (F) GCTAGCTCGAGATGAATGTTGTCGACACTGTCCCATC

exon 16 (R) TCAGGAAGCTTTCTTTCTCAAAGGTCCAGCTTAGATCC

### *In Situs* (LNA, whole mount, and section)

For locked-nucleic acid (LNA) *in situ*, the GEISHA project microRNA detection protocol version 1.1 (http://geisha.arizona.edu) was used. For whole mount *in situ*, embryos were harvested and fixed in 4% DEPC PFA in PBS overnight, rinsed in PBS, put through a methanol series, and then stored in 100% methanol at −20°C until used. For section *in situ*, embryos were harvested and fixed in 4% DEPC PFA in PBS overnight and prepared for cryosectioning at 20 µm. *In situ* hybridization was performed with single-stranded digoxigenin labeled riboprobes directed against the following mRNAs: *FGF8* (C. Tabin), *Lmx1a* (K. Millen), *Lmx1b* (C. Tabin), *LNA probe 135a* (Exiqon 39037-01), *Otx2* (C. Tabin), *miR135a2/Rmst probe A* (Genbank AI853140; B. Harfe), *miR135a2/Rmst probe B* (Genbank BI734849; B. Harfe), *Shh* (A. McMahon), and *Wnt1* (A. McMahon).

### Embryonic stem cell differentiation and quantification

An optimized protocol for dopamine neuron differentiation from embryoid body aggregates was modified from motor neuron differentiation protocol [49] and as described below. An ESC line that harbored a *CAG::CreER^T2^* construct and *Dicer1* ^floxed/floxed^ alleles [5] was transiently treated with the FGF inhibitors PD173074 (Sigma, 50 nM as final concentration) from days 2–4 of culture. Then, on day 4 of culture, Smoothened agonist (EMD 566660, 15 nM as final concentration) and mouse recombinant FGF8b protein (Cell Signaling, 100 ng/ML as final concentration) were added for 4 days. Concomitantly, either control solvent (Ethanol) or 4-hydroxy tamoxifen (4OHT) (Sigma, 250∼500 nM as final concentration) was added for 2 days. On day 9, embryoid bodies were fixed in 4% PFA, cryosectioned, and immunolabeled. Images were collected on a Zeiss LSM510 confocal microscope. MetaMorph Software (Molecular Devices) was used to quantify Lmx1a, Lmx1b, and Nkx6.1 levels in Foxa2+ cells of 15 embryoid bodies (5 each from 3 independent experiments, n = 3).

### Luciferase assays

The stem-loop of *mmu-miR-135a-2* (accession MI0000715) plus ∼100 bp 5′ and 3′ flanking sequence was PCR purified from genomic DNA and then cloned into the *pCAG-RFP-int* vector (Addgene plasmid 19822). A fragment of each target gene *3′UTR*, which corresponds to the region predicted to contain a *mmu-miR-135a-2* seed match, was PCR purified from genomic DNA and then cloned into the *pmirGLO* vector (Promega E1330). The predicted *miR135a2* binding site in the *Lmx1b 3′UTR* was directly mutated by using the Phusion site-directed mutagenesis kit (Thermo Scientific) to create constructs with scrambled or deleted binding sites. The success of mutagenesis was confirmed by sequencing. The following primers, including restriction sites, were used for cloning:

135a2 (F) GCTAGGCGATCGCGTGTGCTTTGTGTCCCTTACATGTAGC

135a2 (R) TTAGGACGCGTGACACTCAAGGAACACCAAAGAGG

Lmx1b (F) GGATCTAGATCCATGCAGAGCTCCTACTTTG

Lmx1b (R) CAGTCTAGAAAGTGACTGTCCAAGAGCTCTGGGTC

Lmx1b-M1 (F): TCAGACTCTTCAGACCAATCAGCGGTGCCCTCCCCT

Lmx1b-M1 (R): GCGGGTGGTGGGCTGGGGG

Lmx1b-M2 (F): CGCTCAGACTCTTCAGACTAGGCTACGGTGCCCT

Lmx1b-M2 (R): GGTGGTGGGCTGGGGGGCC

Lmx1b-del (F): ACGGTGCCCTCCCCTCGGCCAGCC

Lmx1b-del (R): GTCTGAAGAGTCTGAGCGGGTGGTGGGCT

Ccnd1 (F): GCTAGGCTAGCGGTCTGCTTGACTTTCCCAACC

Ccnd1 (R): TCAGGCTCGAGTGACAGGACGATCGCCATCAG

Gsk3b (F): GCTAGGCTAGCAAGGATCATGGCAGGATCCCAG

Gsk3b (R): TCAGGCTCGAGTGTGGAGTGGGCAAAGGTGC

Otx2 (F): GCTAGGCTAGCAGGTTTTGTGAAGACCTGTAGAAGC

Otx2 (R): TCAGGCTCGAGTAGGACAATCAGTCGCACAATC

Tcf7l2 (F): GCTAGGCTAGCCTTGCTGTACCTGTATGTCCGTCC

Tcf7l2 (R): TCAGGCTCGAGCACAGGGCAGTTGACTAGGAGGT

Wnt1 (F): GCTAGCTCGAGTTCTGCACGAGTGTCTATGAGGTG

Wnt1 (R): TTAGGGTCGACAAGGGCGCCTATGAGAAGCTG

The plasmids were co-transfected into 24-well plates of HEK293 cells using Lipofectamine 2000 (Invitrogen #11668). After 24 hours, the cells were harvested for a luciferase assay (Promega E1910) and measured with the Clarity Luminescence Microplate Reader (Bio-Tek). Each Firefly reading was normalized to Renilla and replicated 8 times each in 2 separate experiments.

### Generating and testing the *miR135a2OE* transgene

To generate the *135a2OE* transgene, we isolated a 324 bp fragment of genomic DNA that contains the precursor sequence of *miR135a2*, using the *135a2* forward and reverse primers described above, and cloned it into different mammalian expression vectors. Ultimately, after testing four different vectors *in vitro, CAG-loxP-STOPr-loxP-miR135a2-IRESeGFP* was determined to have the least leakiness even though the STOP cassette was in the reverse orientation. The transgene was microinjected into B6SJL at Northwestern University's Transgenic and Targeted Mutagenesis Laboratory. Six founder lines were obtained, and three separate lines (*En1::Cre;135a2OE #2, #3, and #5*) were used for experiments. All three show a very similar phenotype, in terms of overall midbrain size as well as reduced mDA progenitors, thus ruling out the possibility of site-of-integration dependent phenotypes.

To assess the *miR135a* expression levels in different mutant scenarios we performed qRT-PCR (see above). When *En1::Cre* is used to recombine the transgene, we detect a ∼3 fold increase of *miR135a* in 8.75 dpc mutants compared to littermate controls. When *Nes::Cre* is used to recombine the transgene a few days later, we detect modest overexpression of *miR135a* in 11.5 dpc mutants (See Figure S5D), but the quantification in mDAs is complicated by high endogenous levels of *miR135a* in controls at this stage, particularly in cells exiting the ventricular zone throughout the midbrain (See Figure 4K and Figure S1B). Separate analysis indicates that endogenous *miR135a* levels increase ∼8 fold between 8.5 dpc control samples and 11.5 dpc control samples (data not shown). eGFP activity was undetectable from this transgene in all scenarios tested, as it is likely degraded during microRNA processing.

### Generation of sensor transgenics

For the *miR135a2* “sensor” experiment, we designed a transgene, *CAG-eGFP-WPRE-5XmiR135a2rc*, which contained five exact *miR135a2* reverse complement repeats, each separated by a GGCCGGCC spacer. For a control, we designed a similar transgene, *CAG-tdTomato-WPRE* with no complementary *miR135a2* sites in the *3′UTR*. The transgenes were co-injected into BL6SJL embryos and transient transgenics were harvested at 11.5 dpc or 12.5 dpc.

### Mouse husbandry and genotyping

For *Lmx1b* overexpression experiments, male mice harboring an allele in which *Cre recombinase* was knocked in to the *En1 locus* [41] were bred to mice harboring the *Rosa26^Lmx1b/+^* allele [42]. For *Lmx1b* deletion experiments, mice harboring an allele of *Lmx1b* with loxP sites flanking the homeodomain in exons 4–6 were used [45]. *En1::Cre;Lmx1b^F/+^* males were bred to *Lmx1b^F/F^* females to obtain mutant embryos. Both *En1::Cre-* and *En1::Cre+;Lmx1b^F/+^* littermates were used as controls, as no phenotype was observed in heterozygotes. For *miR135a2* overexpression experiments, we bred females harboring the *135a2OE* allele (described above) with males harboring 1) the *En1::Cre* knock-in allele, 2) an allele in which a *GFP-Cre* fusion cassette was knocked in to the *Shh locus* [8] or 3) a *Nes::Cre* transgene, expressed under control of the rat *Nestin* promoter and enhancer [73]. To assess Wnt signaling, we used mice harboring an allele in which destabilized *eGFP* was placed under control of the *Axin2* promoter, exon 1 and intron 1 (*Axin2::d2eGFP*) [43]. For all matings, the morning when a vaginal plug was detected was designated as 0.5 days post coitum (dpc). Mice were maintained and sacrificed according to the protocols approved by the Northwestern University Animal Care and Use Committee.

### Antibody labeling

Embryos were harvested and fixed in 0.2%–4% PFA in PBS for various amounts of time depending upon embryonic ages, and sectioned at 20 µm. Tissue sections were post-fixed in 1%–4% PFA in PBS, rinsed in PBS, antigen retrieved depending on the antibody (Vector Labs H-3301), blocked in 5% donkey serum, 0.1% Triton X-100 in PBS, and incubated overnight at 4°C with primary antibodies diluted in blocking solution: rabbit Active Caspase-3 (Cell Technology; 1∶1000), rat DAT (Santa Cruz; 1∶50), goat Foxa2 (Santa Cruz; 1∶50), rabbit GFP (Molecular Probes; 1∶1500), mouse Islet-1 (Developmental Studies Hybridoma Bank; 1∶100), guinea pig Lmx1a (Y-C. Ma; 1∶20,000), rabbit Lmx1b (custom; 1∶5,000), mouse Nkx6.1 (Developmental Studies Hybridoma Bank; 1∶100), rabbit Nurr1 (Santa Cruz; 1∶500), rabbit Pitx3 (Zymed; 1∶500), and sheep TH (Pel Freeze; 1∶250). Sections were rinsed in PBS and incubated with appropriate Alexa 488, 555, 647 (Molecular Probes) or Cy3 and Cy5 (Jackson Immuno Research) secondary antibodies diluted 1∶250 in blocking solution, rinsed in PBS, covered with DAPI (1 mg/mL; Sigma) in PBS, rinsed in PBS, and coverslipped followed by epifluorescent (Leica) or confocal microscopy (Zeiss LSM 510 META laser scanning or Leica DM6000 CFS). Images were processed in Adobe Photoshop CS2.

### Measurements, neuron counts, and statistical analysis

To measure the intensity of eGFP and tdTomato fluorescence in 11.5 dpc transient transgenic embryos, images of unstained coronal sections were taken with a Leica DM6000 CFS microscope. After alignment in Adobe Photoshop, such that the sections were equally matched along the anterior-posterior axis, a representative rostral, midlevel and caudal section was selected from each embryo. Using the line scan measurement tool in the Leica Application Suite Advanced Fluorescence Software, two lines were drawn in the medial and lateral domain of each section and the mean fluorescence intensity was recorded. For measurements of the third ventricle and overall midbrain area, coronal sections from *En1::Cre;Lmx1bOE, En1::Cre;Lmx1bcKO*, or *En1::Cre;135a2OE* littermates were aligned in Adobe Photoshop, such that they were equally matched along the anterior-posterior axis. For four representative sections, the edges of the ventricular and pial surfaces were traced, and then both the perimeter and area were calculated using the “Analyze” function in NIH ImageJ. To calculate tissue area, the ventricular area was subtracted from the pial area. To determine FP and mDA progenitor domain size, coronal sections from *En1::Cre;Lmx1bOE, En1::Cre;Lmx1bcKO, En1::Cre;135a2OE, Shh::Cre;135a2OE, or Nes::Cre;135a2OE* littermates were immunostained for Foxa2/Lmx1a/Nkx6.1 and then aligned in Adobe Photoshop, such that they were equally matched along the anterior-posterior axis. For every third section, the distances along the ventricular surface from 1) the ventral midline to the dorsal edge of the Lmx1a domain, and 2) the ventral midline to the dorsal edge of the Foxa2 domain were calculated using the “segmented line measure” function in ImageJ. To account for changes in overall midbrain size, the dorsal-ventral extent of the Lmx1a domain was normalized to the extent of the Foxa2 domain by dividing the first measurement by the second. To quantify the amount of different ventral neuron types in *En1::Cre;Lmx1bOE, En1::Cre;Lmx1bcKO*, and *En1::Cre;135a2OE* littermates, coronal sections were immunostained with combinations of Brn3a/Islet/Lmx1a/TH and then aligned in Adobe Photoshop, such that they were equally matched along the anterior-posterior axis. One-half of a representative rostral, midlevel and caudal section were chosen for manual counting from each embryo, except where noted below. Islet was counted from every third section in *En1::Cre;Lmx1bOE* and *En1::Cre;135a2OE*, and Brn3a was counted from every sixth section in *En1::Cre;Lmx1bOE*. All statistical values were calculated using a two-sample equal variance, two tailed, Student's t-Test.

### Bioinformatic analysis

To determine *miR135a* targets, we used TargetScan, MicroCosm, miRanda, and miRDB algorithms. Wnt pathway genes were obtained from the following references [13], [74], [75].

## Supporting Information

Figure S1 (A) RT-PCR performed on 11.5 dpc ventral midbrain. The fragments produced from the exon 12/exon 16 primer pair revealed that extended variants of the *Rmst* transcript exist. The fragment produced from the exon 15/exon 16 primer pair confirms the presence of the bioinformatically predicted exons that flank *miR135a2*. (B) *In situ* hybridization at 11.5 dpc shows that the *miR135a2/Rmst* probes, *probe A* and *probe B*, have identical expression patterns and are found in the same regions as the *135a LNA* probe (see Figure 1B). On coronal sections that were stained with NBT/BCIP for a short period of time, both *probe A* and *probe B* were detected in the midbrain RP and FP. After longer staining, both *probe A* and *probe B* were also visible, at a lower level, in cells exiting from the midbrain ventricular zone (black arrowheads). Further, both *probe A* and *probe B* were detected in the IsO (black arrows) and the hindbrain Floor Plate (hb FP). (C) *Wnt1::Cre, R26::PFwe* embryo shows reporter (nLacZ) expression throughout the midbrain, as a result of early expression of *Wnt1* in the prospective midbrain as seen in Figure 2C.
(TIF)

Figure S2 (A–D) 11.5 dpc *En1::Cre;Lmx1bcKO* embryos show mildly reduced expression of *miR135a2/Rmst* and *Wnt1* in the dorsal midbrain. (E–F) The *Axin2::d2eGFP* transgene was used as a transcriptional readout of canonical Wnt signaling. d2eGFP fluorescence was observed to be very slightly reduced in the dorsal midbrain region. Scale bar represents 100 µM. (G) qRT-PCR performed on 9.75 dpc midbrain of *En1::Cre;Lmx1bOE* or *En1::Cre;Lmx1bcKO* littermates. Consistent with *miR135a2/Rmst in situ* hybridizations (see Figure 4), *En1::Cre;Lmx1bOE* embryos showed a 1.9 fold increase in mature *miR135a* expression (n = 4 controls, 6 mutants; control mean = 1±0.08, mutant mean = 1.91±0.12; p-value = 0.01). *En1::Cre;Lmx1bcKO* embryos showed a reduction of *miR135a*, although this change did not reach statistical significance (n = 5 controls, 3 mutants; control mean = 1±0.05, mutant mean = 0.81±0.02; p-value = 0.14).
(TIF)

Figure S3 (A–B) In *En1::Cre;Lmx1bOE* embryos, *in situ* hybridization shows that *Wnt1* expression is already increased in the midbrain at 9.5 dpc. Ectopic *Wnt1* is seen most prominently in the dorsal midbrain, but is also visible in ventral-lateral progenitors. (C–D) Immunostaining at 10.5 dpc shows that the DV extent of both Foxa2 and Lmx1a is expanded. (E–F) 11.5 dpc immunolabeling demonstrates the overexpression of Lmx1b within mDA progenitors. Measurement of the third ventricle (3V) at 11.5 dpc revealed a 57% increase in size (n = 3; control mean = 1971±56.88 µM, mutant mean = 3092±110.35 µM; p-value = 0.0008). (G–H) 14.5 dpc coronal sections show the increased size and morphogenetic changes in the *En1::Cre;Lmx1bOE* mutant midbrain. Alkaline phosphatase (AP) histochemistry shows widespread expression of the *Lmx1b-IRES-AP* transgene using *En1::Cre*. (I–N) Immunostaining of 14.5 dpc *En1::Cre;Lmx1bOE* embryos shows that in rostral sections, Nurr1+ cells were present medially, but there appears to be a reduction in medially located TH+ neurons (white asterisks). This Nurr1+/TH− phenotype is possibly because of increased *Wnt1*/Wnt signaling (Joksimovic, unpublished observations). (O–R) At mid- and caudal- levels in these embryos, ectopic Nurr1+/TH+ neurons were observed in the lateral regions (yellow arrows). Scale bars represent 100 µM.
(TIF)

Figure S4 (A–B) Activated Caspase-3 immunostaining of 9.5 dpc *En1::Cre;Lmx1bcKO* midbrain revealed increased apoptosis, predominantly in lateral regions, compared to controls (neural tissue was outlined in white). (C–H) Foxa2 and Lmx1a immunostaining showed a reduction in the DV extent of the FP and mDA progenitor domain by 9.5 dpc. (I–J) A few Islet+ cells were detected in the mutant midbrain at this stage, though they could not be detected in 13.5 dpc mutants. (K–L) 11.5 dpc *in situ* hybridizations show that *Otx2* expression appears decreased in the *En1::Cre;Lmx1bcKO* mutant midbrain. Further, measurement of the ventricular perimeter revealed a 30.2% reduction in size in mutants (n = 3; control mean = 1732.28±5.80 µM, mutant mean = 1209.72±34.07 µM; p-value = 0.0001). (M–N) Analysis of the midbrain/hindbrain junction revealed that relative to controls (*En1::Cre−;Lmx1b^F/F^ or En1::Cre+;Lmx1b^F/+^*) some *Otx2+* cells appear to have crossed the isthmic boundary into the hindbrain (arrowhead), and (O–P) *Fgf8* was abolished. Scale bars represent 100 µM.
(TIF)

Figure S5 (A–C) Immunostaining from separate 13.5 dpc *En1::Cre;135a2OE* transgenic lines showed similar reduction of Lmx1a/TH+ mDA neurons, thus ruling out the possibility of site-of-integration dependent phenotypes. (D) qRT-PCR demonstrates overexpression of the *CAG-loxP-STOPr-loxP-miR135a2-IRESeGFP* transgene using *En1::Cre* or *Nes::Cre*. In 8.5 dpc *En1::Cre;135a2OE* mutant heads there was a 3.03 fold increase in *miR135a* levels compared to transgene negative controls (n = 4 controls, 12 mutants; control mean = 1±0.06, mutant mean = 3.03±0.18; p-value = 0.02). In 11.5 dpc *Nes::Cre;135a2OE* mutant midbrain there was a 1.35 fold increase in *miR135a* levels compared to littermate controls (n = 7; control mean = 1±0.17, mutant mean = 1.35±0.18; p-value = 0.18). The increase detected did not reach statistical significance, likely due to the fact that in 11.5 dpc midbrain the endogenous net *miR135a2* levels have increased because of miR expression in cells exiting the ventricular zone throughout the DV axis (see Figure 4K and Figure S1B).
(TIF)

Figure S6 (A–B) Activated Caspase-3 immunostaining of 9.5 dpc *En1::Cre;135a2OE* midbrain, revealed an increase in the number of apoptotic cells compared to controls. Note that apoptotic cells are less prevalent in the ventral midbrain. (neural tissue was outlined in white). (C–H) Foxa2 and Lmx1a immunostaining showed a reduction in the DV extent of the FP and mDA progenitor domain by 9.5 dpc. (I) qRT-PCR performed on 8.5 dpc heads of *En1::Cre; 135a2OE* littermates showed a 63% reduction in *Otx2* expression (n = 5 controls, 7 mutants; control mean = 1±0.11, mutant mean = 0.37±0.03; p-value = 0.059). (J–K) Consistent with the qRT-PCR, 11.5 dpc *in situ* hybridizations show that *Otx2* expression is decreased in the *En1::Cre; 135a2OE* mutant midbrain. Further, measurement of the ventricular perimeter of coronal sections revealed a 12.6% reduction in size (n = 6; control mean = 1859.22±71.25 µM, mutant mean = 1624.42±33.5 µM; p-value = 0.01) in mutants. (L–O) Immunostaining showed that TH+ mDAs and Islet+ oculomotor neurons were reduced in numbers in 11.5 dpc *En1::Cre;135a2OE* mutants (sections were outlined to accentuate tissue). (P–Q) 12.5 dpc *in situ* hybridization shows particularly severe *En1::Cre;135a2OE* mutant, in which *Shh* expression is maintained at the midline, similar to *En1::Cre;Lmx1bcKO* mutants. Scale bars represent 100 µM.
(TIF)

Figure S7 (A) Several molecules involved in the canonical Wnt signaling pathway, including *Wnt1* itself, are predicted targets of *miR135a* (microrna.org)(the *miR135a* seed is highlighted in blue and corresponding binding sites in the target genes are highlighted in yellow). (B) Transient transfection in HEK293 cells showed that *miR135a2* was sufficient to repress constructs containing fragments of the *Ccnd1* (empty mean = 1±0.13, *miR135a2* mean = 0.77±0.07; p-value = 0.0002), *Gsk3b* (empty mean = 1±0.07, *miR135a2* mean = 0.62±0.05; p-value = 1.53E-07), *and Tcf7l2* (empty mean = 1±0.06, *miR135a2* mean = 0.73±0.07; p-value = 1.06E-05) *3′UTRs. Otx2* (empty mean = 1±0.08, *miR135a2* mean = 0.91±0.09; p-value = 0.10) and *Wnt1* (empty mean = 1±0.13, *miR135a2* mean = 0.87±0.14; p-value = 0.37) *3′UTRs* were mildly repressed, but did not reach statistical significance (n = 8 each in two separate experiments).
(TIF)

Figure S8 (A–F) Analysis of the midbrain/hindbrain junction at 11.5 dpc revealed that the Lmx1b, *Wnt1*, and *Fgf8* domains were narrower in *En1::Cre;135a2OE* mutants. (G–H) The isthmic boundary, as determined by *Otx2*, was unchanged, although the isthmic constriction appeared less prominent.
(TIF)

Figure S9 (A–D) Analysis of 13.5 dpc *En1::Cre;135a2OE* embryos revealed that the total number of Brn3a+ neurons, which derive from Nkx6.1+ progenitors, was not drastically altered. Additionally, several Brn3a+ cells were observed at the midline of the dopaminergic field (red asterisks), similar to the *En1::Cre;Lmx1bcKO*.
(TIF)

Figure S10 Comparison of *Lmx1bcKO* and *135a2OE* phenotypes revealed a striking similarity between the mutants. The *En1::Cre;Lmx1bcKO* is more affected than the *En1::Cre;135a2OE* in several criteria tested, likely because *Lmx1b* levels are not completely abolished in *En1::Cre;135a2OE* mutants. These data suggest that the *En1::Cre;135a2OE* phenotype is at least in part dependent on *Lmx1b* repression in the early embryo.
(TIF)
