# Supplementary figures and images for: An Lmx1b-miR135a2 Regulatory Circuit Modulates Wnt1/Wnt Signaling and Determines the Size of the Midbrain Dopaminergic Progenitor Pool
Source: PLoS Genet. 2013 Dec 12;9(12):e1003973. doi: 10.1371/journal.pgen.1003973 (PMC3861205; doi:10.1371/journal.pgen.1003973)

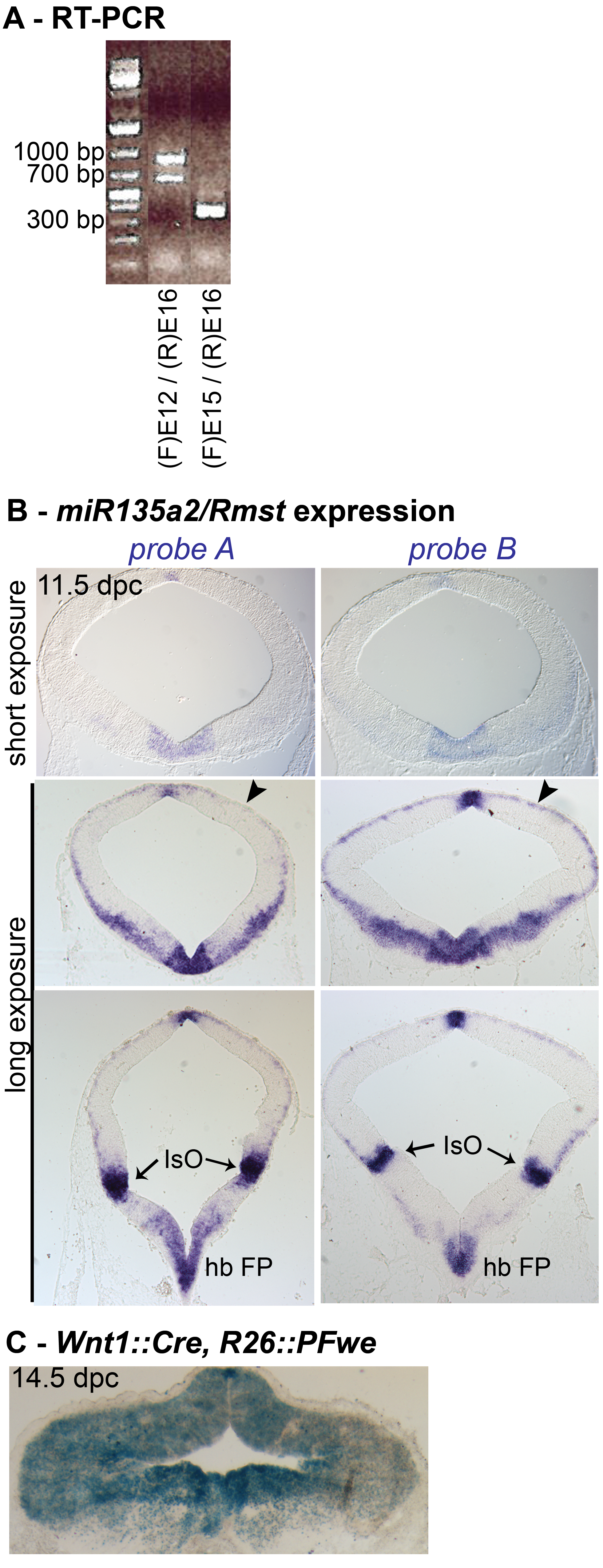

Supplement: Figure S1 — (A) RT-PCR performed on 11.5 dpc ventral midbrain. The fragments produced from the exon 12/exon 16 primer pair revealed that extended variants of the Rmst transcript exist. The fragment produced from the exon 15/exon 16 primer pair confirms the presence of the bioinformatically predicted exons that flank miR135a2. (B) In situ hybridization at 11.5 dpc shows that the miR135a2/Rmst probes, probe A and probe B, have identical expression patterns and are found in the same regions as the 135a LNA probe (see Figure 1B). On coronal sections that were stained with NBT/BCIP for a short period of time, both probe A and probe B were detected in the midbrain RP and FP. After longer staining, both probe A and probe B were also visible, at a lower level, in cells exiting from the midbrain ventricular zone (black arrowheads). Further, both probe A and probe B were detected in the IsO (black arrows) and the hindbrain Floor Plate (hb FP). (C) Wnt1::Cre, R26::PFwe embryo shows reporter (nLacZ) expression throughout the midbrain, as a result of early expression of Wnt1 in the prospective midbrain as seen in Figure 2C. (TIF) [file pgen.1003973.s001.tif]

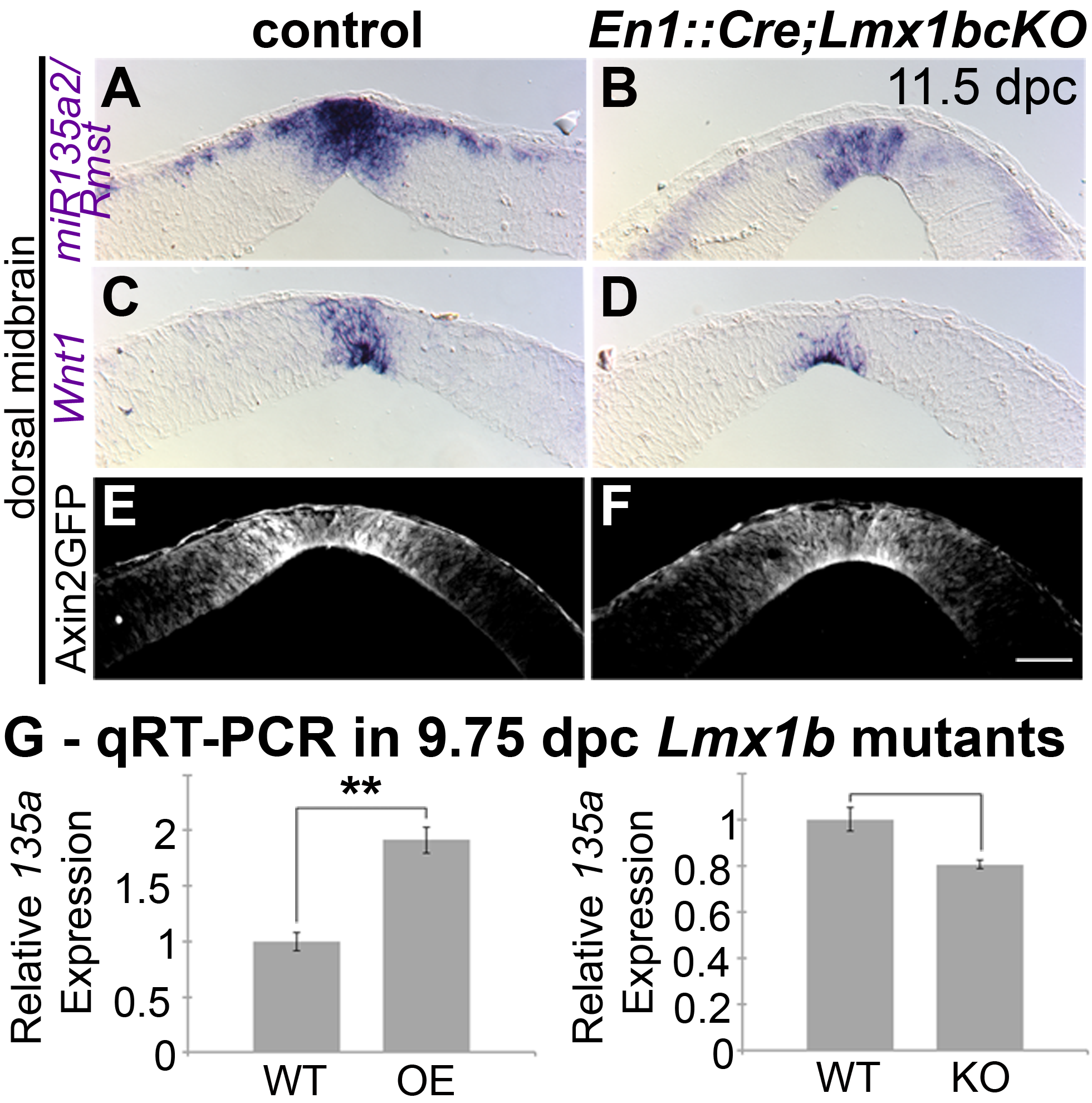

Supplement: Figure S2 — (A–D) 11.5 dpc En1::Cre;Lmx1bcKO embryos show mildly reduced expression of miR135a2/Rmst and Wnt1 in the dorsal midbrain. (E–F) The Axin2::d2eGFP transgene was used as a transcriptional readout of canonical Wnt signaling. d2eGFP fluorescence was observed to be very slightly reduced in the dorsal midbrain region. Scale bar represents 100 µM. (G) qRT-PCR performed on 9.75 dpc midbrain of En1::Cre;Lmx1bOE or En1::Cre;Lmx1bcKO littermates. Consistent with miR135a2/Rmst in situ hybridizations (see Figure 4), En1::Cre;Lmx1bOE embryos showed a 1.9 fold increase in mature miR135a expression (n = 4 controls, 6 mutants; control mean = 1±0.08, mutant mean = 1.91±0.12; p-value = 0.01). En1::Cre;Lmx1bcKO embryos showed a reduction of miR135a, although this change did not reach statistical significance (n = 5 controls, 3 mutants; control mean = 1±0.05, mutant mean = 0.81±0.02; p-value = 0.14). (TIF) [file pgen.1003973.s002.tif]

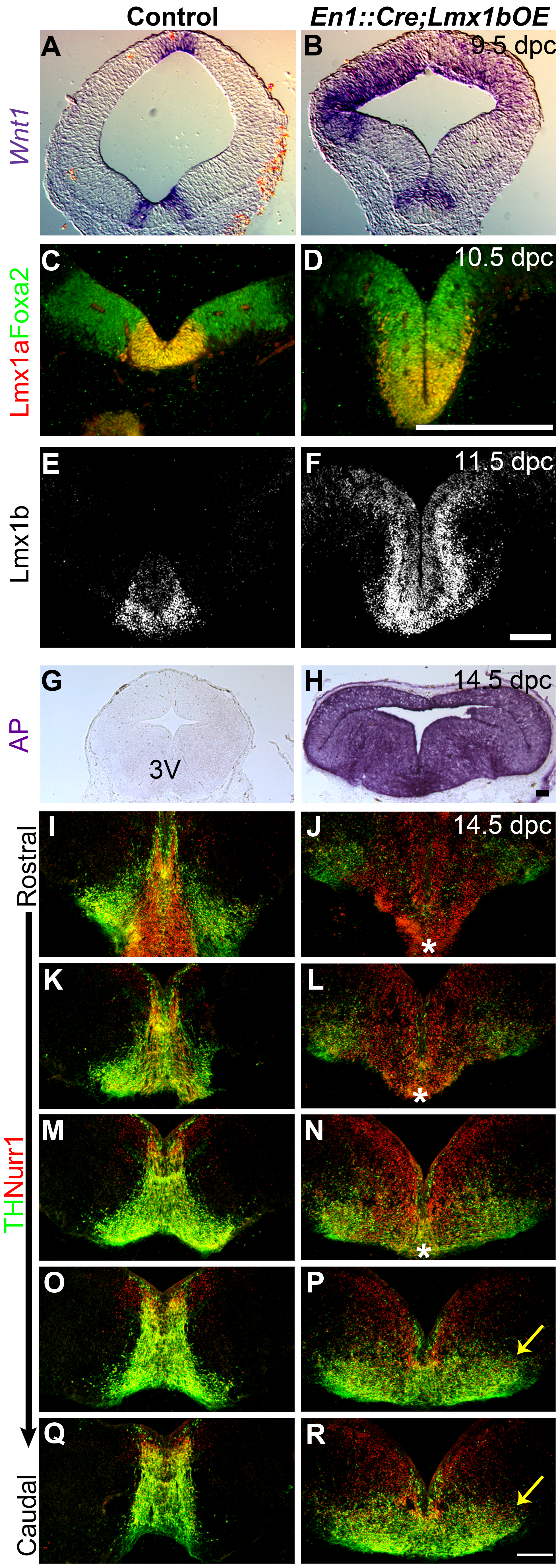

Supplement: Figure S3 — (A–B) In En1::Cre;Lmx1bOE embryos, in situ hybridization shows that Wnt1 expression is already increased in the midbrain at 9.5 dpc. Ectopic Wnt1 is seen most prominently in the dorsal midbrain, but is also visible in ventral-lateral progenitors. (C–D) Immunostaining at 10.5 dpc shows that the DV extent of both Foxa2 and Lmx1a is expanded. (E–F) 11.5 dpc immunolabeling demonstrates the overexpression of Lmx1b within mDA progenitors. Measurement of the third ventricle (3V) at 11.5 dpc revealed a 57% increase in size (n = 3; control mean = 1971±56.88 µM, mutant mean = 3092±110.35 µM; p-value = 0.0008). (G–H) 14.5 dpc coronal sections show the increased size and morphogenetic changes in the En1::Cre;Lmx1bOE mutant midbrain. Alkaline phosphatase (AP) histochemistry shows widespread expression of the Lmx1b-IRES-AP transgene using En1::Cre. (I–N) Immunostaining of 14.5 dpc En1::Cre;Lmx1bOE embryos shows that in rostral sections, Nurr1+ cells were present medially, but there appears to be a reduction in medially located TH+ neurons (white asterisks). This Nurr1+/TH− phenotype is possibly because of increased Wnt1/Wnt signaling (Joksimovic, unpublished observations). (O–R) At mid- and caudal- levels in these embryos, ectopic Nurr1+/TH+ neurons were observed in the lateral regions (yellow arrows). Scale bars represent 100 µM. (TIF) [file pgen.1003973.s003.tif]

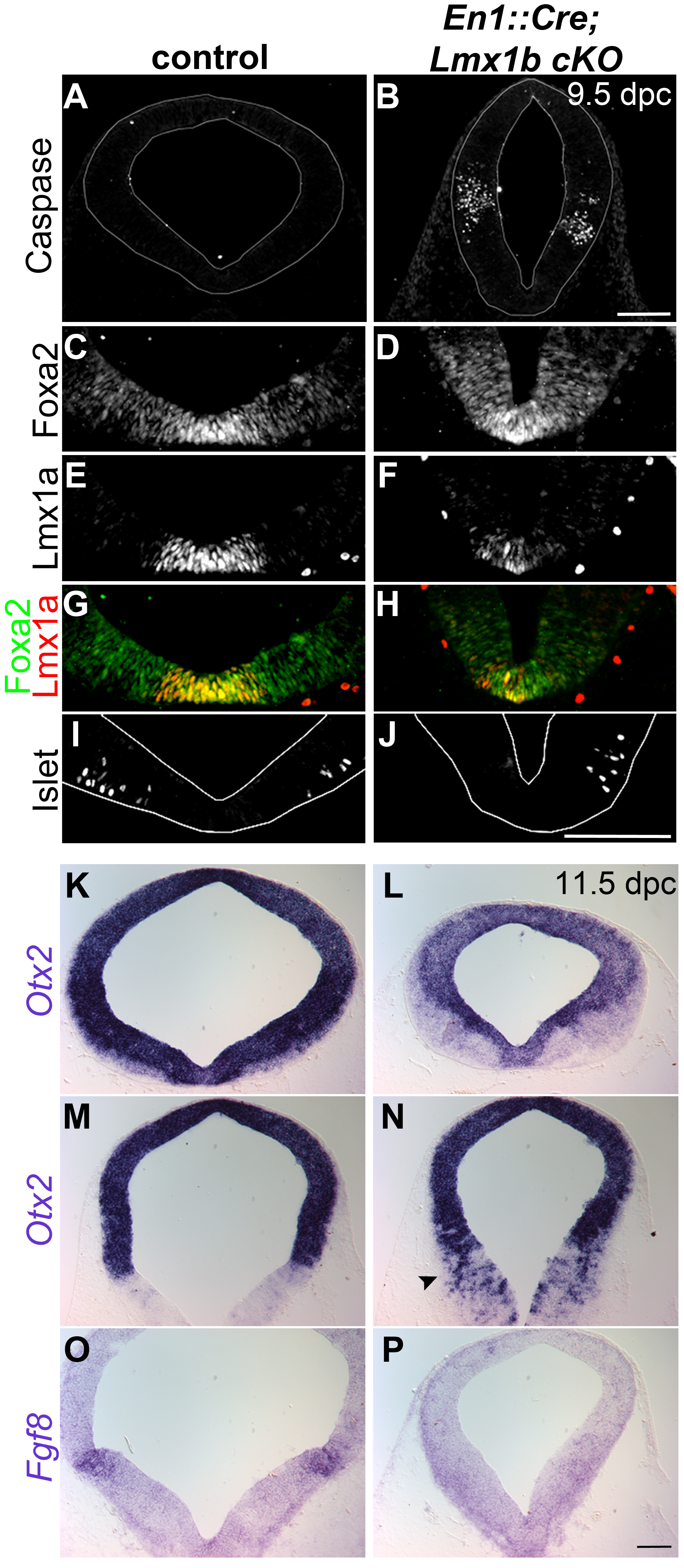

Supplement: Figure S4 — (A–B) Activated Caspase-3 immunostaining of 9.5 dpc En1::Cre;Lmx1bcKO midbrain revealed increased apoptosis, predominantly in lateral regions, compared to controls (neural tissue was outlined in white). (C–H) Foxa2 and Lmx1a immunostaining showed a reduction in the DV extent of the FP and mDA progenitor domain by 9.5 dpc. (I–J) A few Islet+ cells were detected in the mutant midbrain at this stage, though they could not be detected in 13.5 dpc mutants. (K–L) 11.5 dpc in situ hybridizations show that Otx2 expression appears decreased in the En1::Cre;Lmx1bcKO mutant midbrain. Further, measurement of the ventricular perimeter revealed a 30.2% reduction in size in mutants (n = 3; control mean = 1732.28±5.80 µM, mutant mean = 1209.72±34.07 µM; p-value = 0.0001). (M–N) Analysis of the midbrain/hindbrain junction revealed that relative to controls (En1::Cre−;Lmx1bF/F or En1::Cre+;Lmx1bF/+) some Otx2+ cells appear to have crossed the isthmic boundary into the hindbrain (arrowhead), and (O–P) Fgf8 was abolished. Scale bars represent 100 µM. (TIF) [file pgen.1003973.s004.tif]

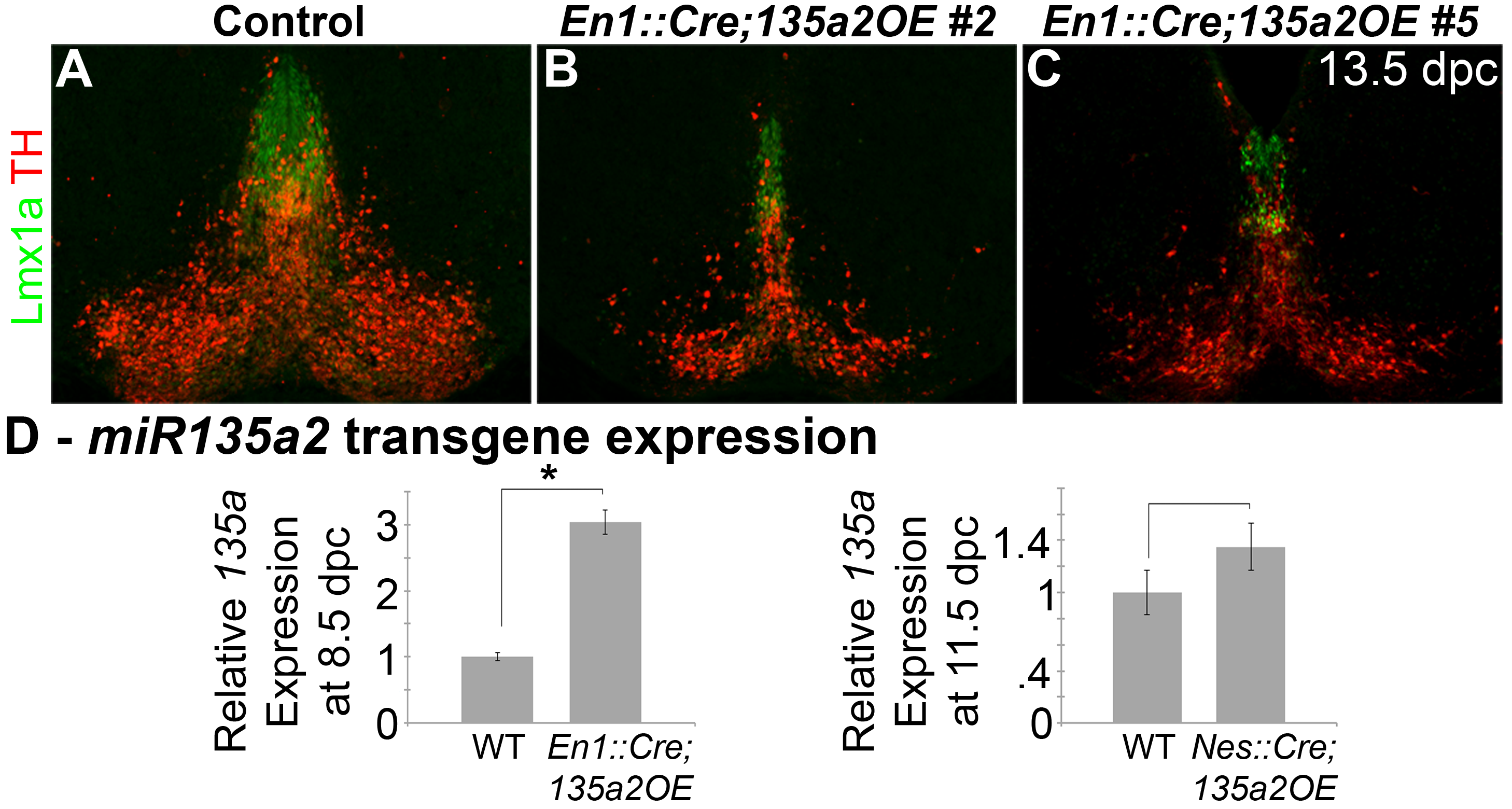

Supplement: Figure S5 — (A–C) Immunostaining from separate 13.5 dpc En1::Cre;135a2OE transgenic lines showed similar reduction of Lmx1a/TH+ mDA neurons, thus ruling out the possibility of site-of-integration dependent phenotypes. (D) qRT-PCR demonstrates overexpression of the CAG-loxP-STOPr-loxP-miR135a2-IRESeGFP transgene using En1::Cre or Nes::Cre. In 8.5 dpc En1::Cre;135a2OE mutant heads there was a 3.03 fold increase in miR135a levels compared to transgene negative controls (n = 4 controls, 12 mutants; control mean = 1±0.06, mutant mean = 3.03±0.18; p-value = 0.02). In 11.5 dpc Nes::Cre;135a2OE mutant midbrain there was a 1.35 fold increase in miR135a levels compared to littermate controls (n = 7; control mean = 1±0.17, mutant mean = 1.35±0.18; p-value = 0.18). The increase detected did not reach statistical significance, likely due to the fact that in 11.5 dpc midbrain the endogenous net miR135a2 levels have increased because of miR expression in cells exiting the ventricular zone throughout the DV axis (see Figure 4K and Figure S1B). (TIF) [file pgen.1003973.s005.tif]

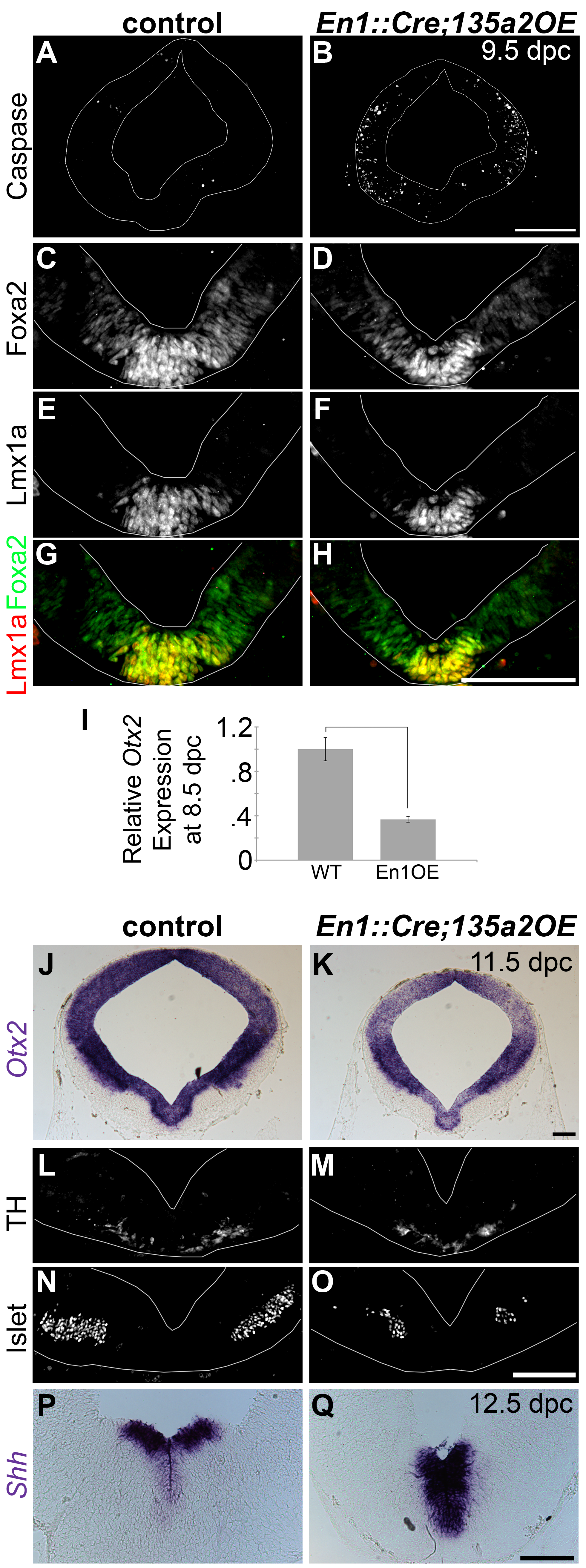

Supplement: Figure S6 — (A–B) Activated Caspase-3 immunostaining of 9.5 dpc En1::Cre;135a2OE midbrain, revealed an increase in the number of apoptotic cells compared to controls. Note that apoptotic cells are less prevalent in the ventral midbrain. (neural tissue was outlined in white). (C–H) Foxa2 and Lmx1a immunostaining showed a reduction in the DV extent of the FP and mDA progenitor domain by 9.5 dpc. (I) qRT-PCR performed on 8.5 dpc heads of En1::Cre; 135a2OE littermates showed a 63% reduction in Otx2 expression (n = 5 controls, 7 mutants; control mean = 1±0.11, mutant mean = 0.37±0.03; p-value = 0.059). (J–K) Consistent with the qRT-PCR, 11.5 dpc in situ hybridizations show that Otx2 expression is decreased in the En1::Cre; 135a2OE mutant midbrain. Further, measurement of the ventricular perimeter of coronal sections revealed a 12.6% reduction in size (n = 6; control mean = 1859.22±71.25 µM, mutant mean = 1624.42±33.5 µM; p-value = 0.01) in mutants. (L–O) Immunostaining showed that TH+ mDAs and Islet+ oculomotor neurons were reduced in numbers in 11.5 dpc En1::Cre;135a2OE mutants (sections were outlined to accentuate tissue). (P–Q) 12.5 dpc in situ hybridization shows particularly severe En1::Cre;135a2OE mutant, in which Shh expression is maintained at the midline, similar to En1::Cre;Lmx1bcKO mutants. Scale bars represent 100 µM. (TIF) [file pgen.1003973.s006.tif]

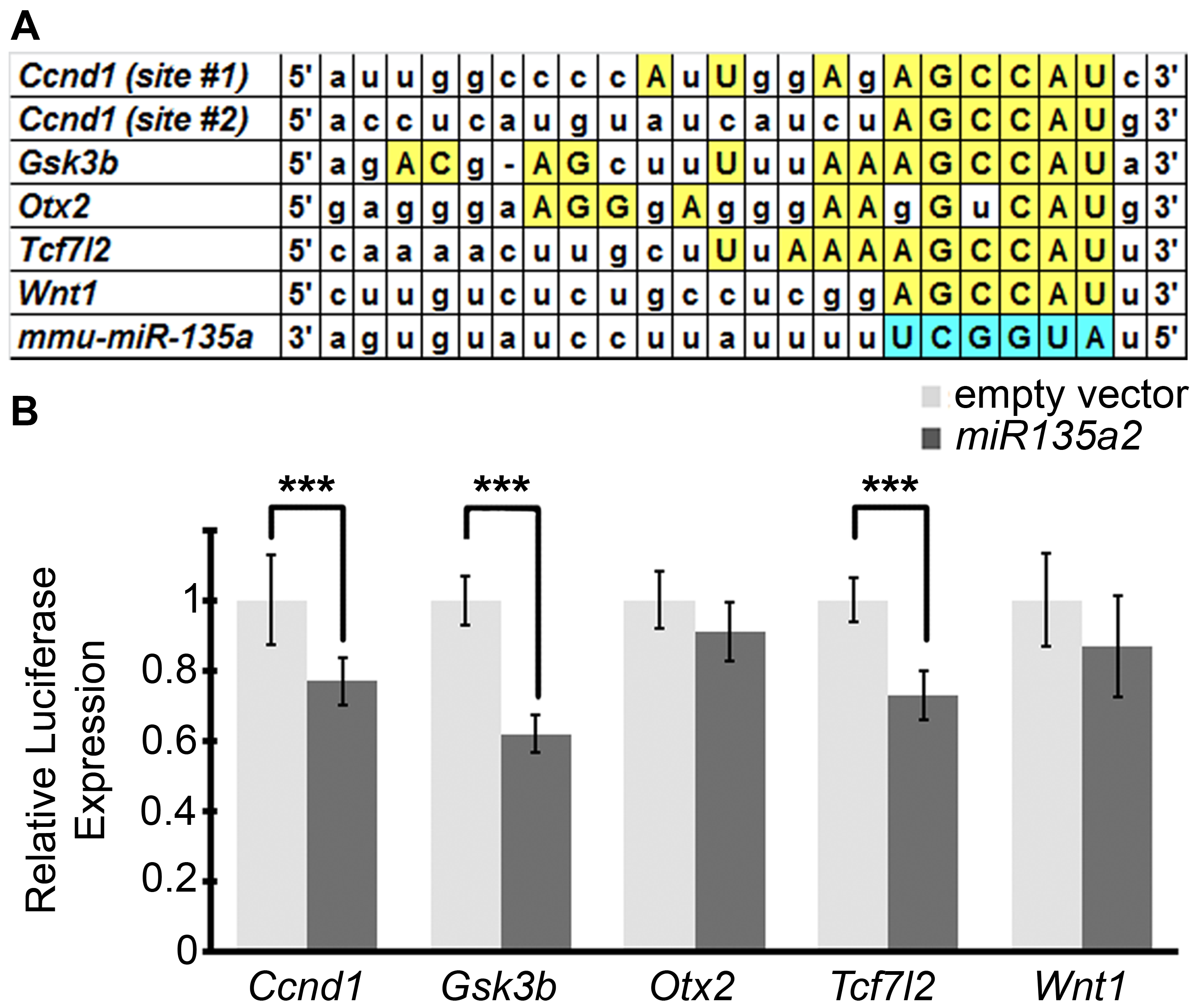

Supplement: Figure S7 — (A) Several molecules involved in the canonical Wnt signaling pathway, including Wnt1 itself, are predicted targets of miR135a (microrna.org)(the miR135a seed is highlighted in blue and corresponding binding sites in the target genes are highlighted in yellow). (B) Transient transfection in HEK293 cells showed that miR135a2 was sufficient to repress constructs containing fragments of the Ccnd1 (empty mean = 1±0.13, miR135a2 mean = 0.77±0.07; p-value = 0.0002), Gsk3b (empty mean = 1±0.07, miR135a2 mean = 0.62±0.05; p-value = 1.53E-07), and Tcf7l2 (empty mean = 1±0.06, miR135a2 mean = 0.73±0.07; p-value = 1.06E-05) 3′UTRs. Otx2 (empty mean = 1±0.08, miR135a2 mean = 0.91±0.09; p-value = 0.10) and Wnt1 (empty mean = 1±0.13, miR135a2 mean = 0.87±0.14; p-value = 0.37) 3′UTRs were mildly repressed, but did not reach statistical significance (n = 8 each in two separate experiments). (TIF) [file pgen.1003973.s007.tif]

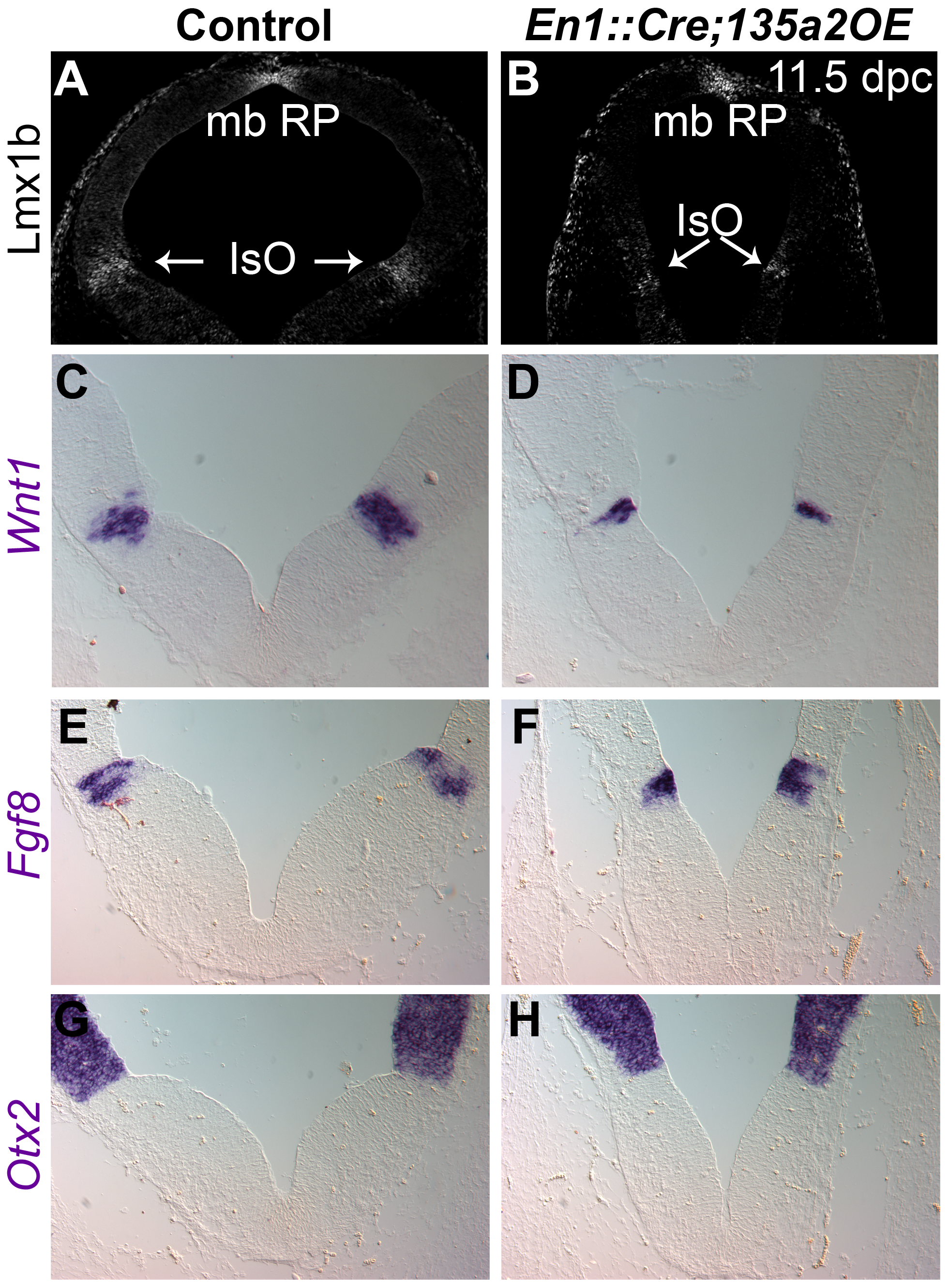

Supplement: Figure S8 — (A–F) Analysis of the midbrain/hindbrain junction at 11.5 dpc revealed that the Lmx1b, Wnt1, and Fgf8 domains were narrower in En1::Cre;135a2OE mutants. (G–H) The isthmic boundary, as determined by Otx2, was unchanged, although the isthmic constriction appeared less prominent. (TIF) [file pgen.1003973.s008.tif]

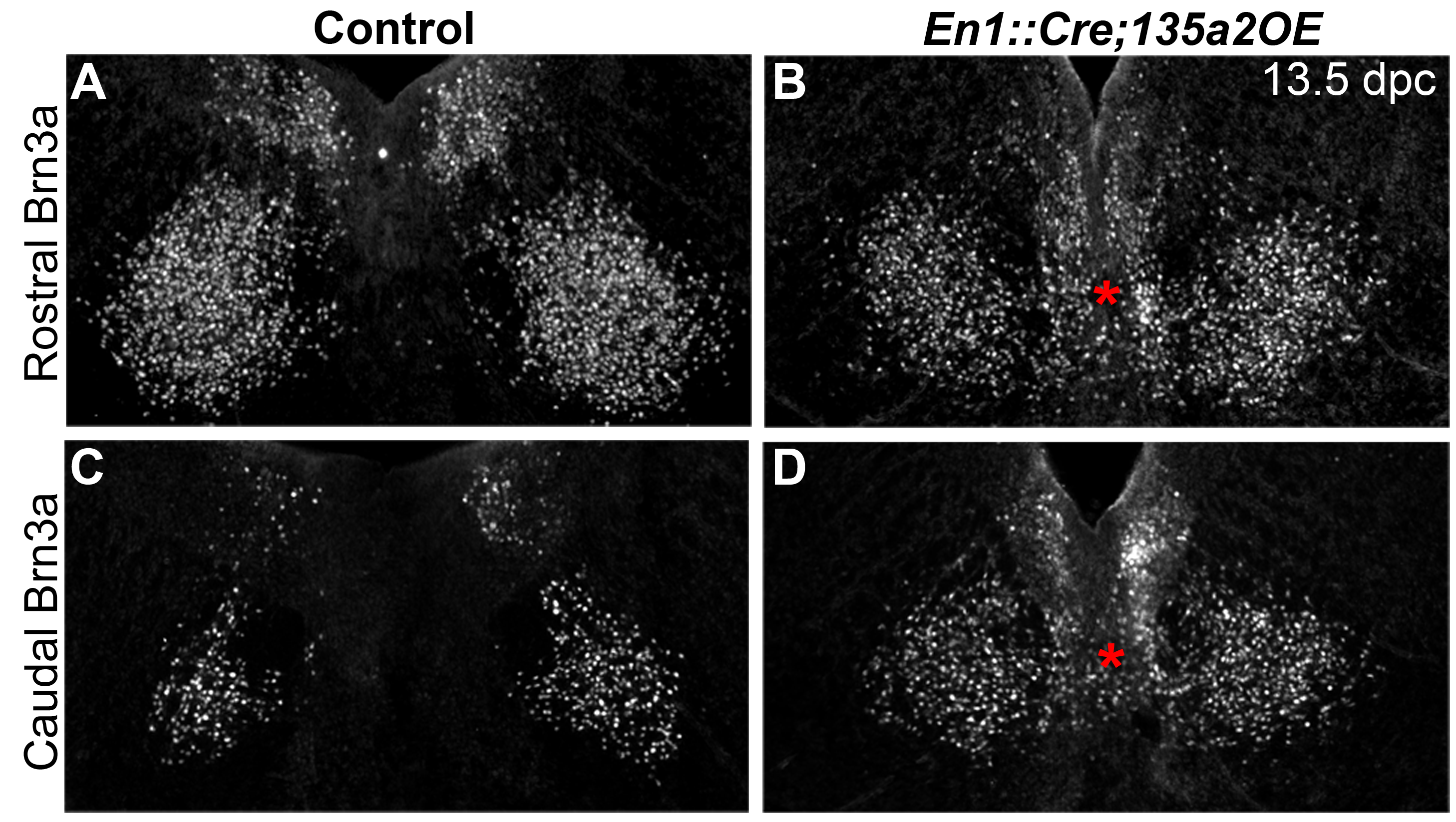

Supplement: Figure S9 — (A–D) Analysis of 13.5 dpc En1::Cre;135a2OE embryos revealed that the total number of Brn3a+ neurons, which derive from Nkx6.1+ progenitors, was not drastically altered. Additionally, several Brn3a+ cells were observed at the midline of the dopaminergic field (red asterisks), similar to the En1::Cre;Lmx1bcKO. (TIF) [file pgen.1003973.s009.tif]

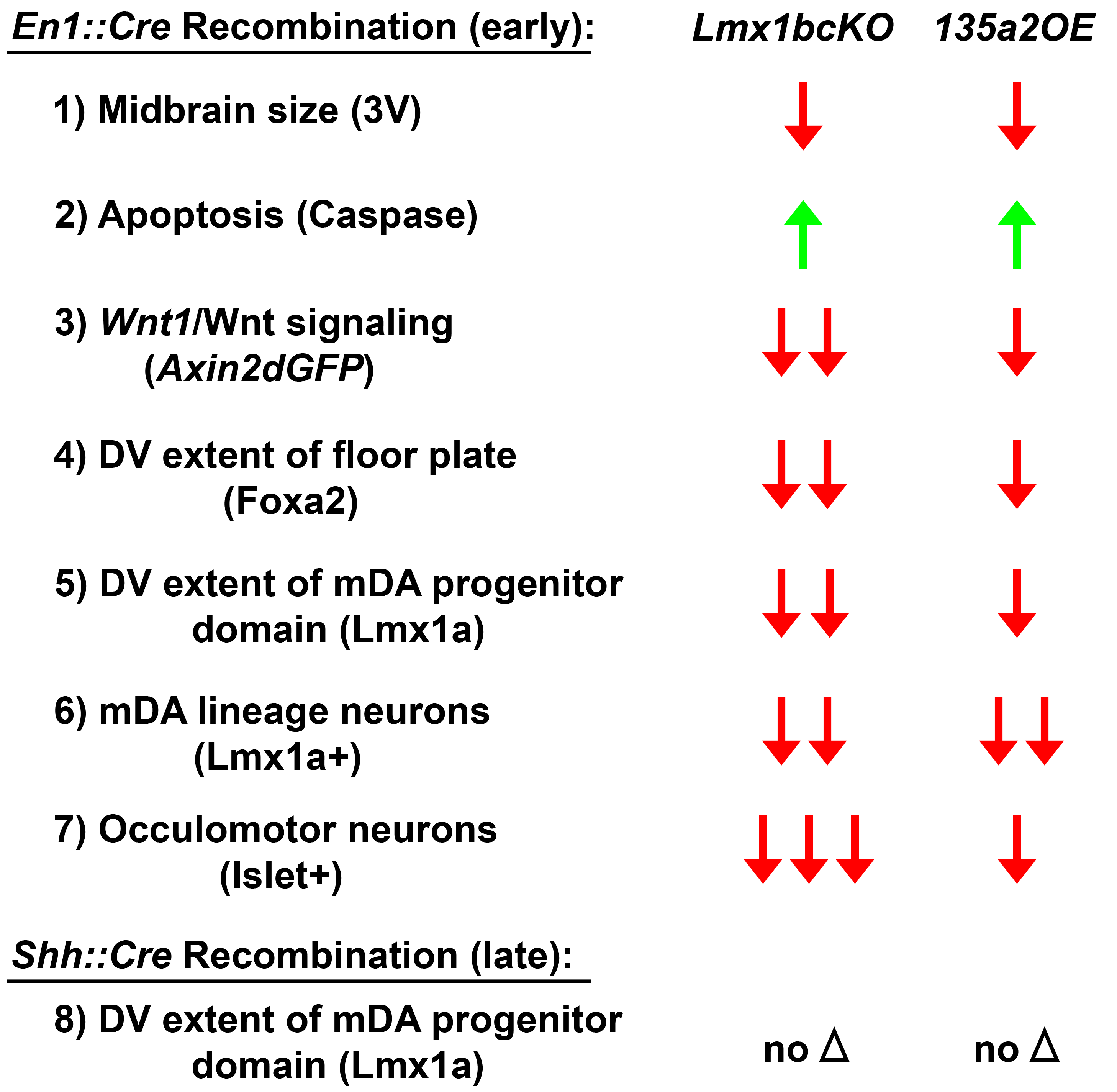

Supplement: Figure S10 — Comparison of Lmx1bcKO and 135a2OE phenotypes revealed a striking similarity between the mutants. The En1::Cre;Lmx1bcKO is more affected than the En1::Cre;135a2OE in several criteria tested, likely because Lmx1b levels are not completely abolished in En1::Cre;135a2OE mutants. These data suggest that the En1::Cre;135a2OE phenotype is at least in part dependent on Lmx1b repression in the early embryo. (TIF) [file pgen.1003973.s010.tif]
